# Dye-sensitized cascaded energy transfer for amplified 1525 nm luminescence in highly doped lanthanide nanoparticles

**DOI:** 10.1038/s41377-026-02302-9

**Published:** 2026-04-27

**Authors:** Fei Long, Dechao Gan, Haoran Chen, Qiqing Li, Wang Wang, Zexuan Sui, Youlin Zhang, Dabing Li, Yulei Chang

**Affiliations:** 1 State Key Laboratory of Luminescence Science and Technology, Changchun Institute of Optics, Fine Mechanics and Physics, Chinese Academy of Sciences, Changchun, 130033 China; 2 University of the Chinese Academy of Sciences, Beijing, 100049 China; 3 College of Physics and Information Engineering, Fuzhou University, Fuzhou, 350108 China; 4 School of Chemistry and Pharmaceutical Sciences, Guangxi Normal University, Guilin, 541000 China

**Keywords:** Nanoparticles, Imaging and sensing

## Abstract

Lanthanide-based probes for second near-infrared (NIR-II) luminescence imaging enable deep-tissue penetration with minimal autofluorescence. However, their broader application is hindered by intrinsic limitations such as low brightness and weak absorption. To address these, we developed a dye-sensitized construct, NaErF_4_@NaYF_4_:50%Yb@ICG. This design harnesses population dynamics in heavily doped Er^3+^ systems through a cascaded energy transfer process enabled by dual 808 nm excitation of both indocyanine green (ICG) and the Er^3+^-rich core—specifically, it harvests energy destined for nonradiative decay by inserting a Yb^3+^-mediated relay (ICG → Yb^3+^ → Er^3+^) into the original ICG → Er^3+^ pathway. This approach yields 1965-fold and 11-fold enhancements in 1525 nm downshifting emission compared to the corresponding core and counterpart, respectively. The resulting nanoprobe enables high-resolution NIR-IIb vascular imaging with a signal-to-background ratio of 3.09. These mechanistic insights and design principles inform the design of efficient NIR-II nanoprobes, demonstrating substantial potential for advancing vascular biology.

## Abstract (web summary)

By harvesting the energy destined for nonradiative decay and redirecting energy flux in dye-sensitized Er^3+^-based nanoparticles via an active shell (Yb^3+^), a three-order-of-magnitude enhancement in NIR-IIb emission was achieved.

## Graphical abstract

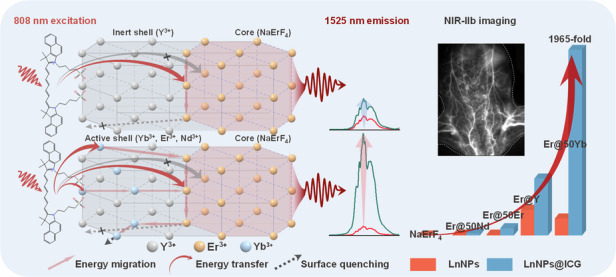

## Introduction

Fluorescence/luminescence imaging represents a novel and promising visualization approach, offering advantages in terms of invasiveness and real-time feedback, and has generated significant scientific and practical interest in preclinical research, including disease diagnosis and monitoring therapeutic effects^1–6^. However, the image quality is often compromised by tissue photon scattering, absorption, and autofluorescence, which introduce background interference and spectral crosstalk^7,8^. The second near-infrared (NIR-II) window (1000–2000 nm) has emerged as a powerful solution to mitigate these limitations. Although sub-windows such as NIR-IIa (1300–1400 nm) already offer advantages over NIR-I, the NIR-IIb (1500–1700 nm) and NIR-IIc (1700–2000 nm) regions provide further reduced scattering and tissue autofluorescence, enabling deeper tissue penetration and higher signal-to-background ratios than both NIR-I and the shorter NIR-II sub-windows^9–12^. Among the various excitation-emission pairs within this window, the combination of ~808 nm excitation and >1500 nm emission is desirable due to its deep-tissue penetration, minimal water-heating, and the commercial availability of efficient 808 nm lasers, as well as the significantly reduced tissue scattering achieved in the NIR-IIb sub-window. To leverage this advantage, a variety of NIR-IIb probes have been developed, including quantum dots^13,14^, aggregation-induced emission luminogens^15–17^, and lanthanide-doped nanoparticles (LnNPs)^18–20^. Among these, LnNPs have emerged as a captivating class of luminescent materials that offer narrow absorption/emission bands, high photostability, long luminescence lifetimes, and large spectral shifts^21–23^. In particular, Er^3+^-based nanostructures are premier candidates, prized for their characteristic, intense emission at ~1530 nm, which aligns with the NIR-IIb subwindow^24,25^.

However, the performance of these systems is fundamentally constrained by the inherently small absorption cross-section of Er^3+^ (*σ* ~ 10^−21 ^cm^2^/ion@808 nm)^26^, a challenge common to many LnNPs due to f-f forbidden transitions. A direct approach to enhance absorption is to increase the Er^3+^ doping concentration, culminating in an Er^3+^-rich system, even a pure 100% Er^3+^-occupied lattice, i.e., NaErF_4_. While this strategy maximizes the number of absorbing and emitting centers, it inevitably intensifies concentration quenching. The construction of a core-shell architecture (e.g., NaErF_4_@NaYF_4_) has been established as an effective means to suppress this quenching^27,28^. Critically, however, even with an optimally engineered core-shell structure, the fundamental limitation of weak 808 nm absorption by Er^3+^ remains, imposing a stringent brightness ceiling.

Dye sensitization, which employs organic dyes with absorption cross-sections orders of magnitude larger than those of lanthanide ions, presents a powerful strategy to overcome this absorption bottleneck^29–32^. This strategy has been widely applied to lanthanide nanoprobes, but almost exclusively to the conventional Yb^3+^/Er^3+^ co‑doped systems (e.g., NaYF_4_:Yb/Er with an inert or @Yb/Nd-containing shell)^33–35^. In these systems, Er^3+^ serves exclusively as the emitter, while Yb^3+^ (or Nd^3+^) mediates absorption and energy migration. By contrast, dye sensitization of Er^3+^-rich cores—particularly in conjunction with active shells—remains largely unexplored. A small number of studies have validated the feasibility of this approach, including the use of 540 nm-excitable thermally activated delayed fluorescence (TADF) dye APDC-DTPA (AD) on NaErF_4_@NaYF_4_^36^, ~635 nm-excitable Cy5 on similar core-shell nanoparticles^37^, and 808 nm-excitable indocyanine green (ICG) on low-doped NaYF_4_:Er systems^38^. Nonetheless, the efficiency of dye sensitization is governed by stringent factors, primarily the distance between the dye donor and the lanthanide acceptor, with effective Förster resonance energy transfer (FRET) typically confined to a short range^39^. This implies that only lanthanide ions near the nanoparticle surface can be efficiently populated via nonradiative energy transfer, while the majority of emitters in the core remain underutilized. Therefore, a fundamental challenge persists: how to maximize the energy transfer efficiency from the surface-bound dye to the entire heavily doped lanthanide core.

In this study, we propose an active-shell engineering strategy designed to enhance energy transfer efficiency in dye-sensitized Er^3+^-rich nanoprobes. We demonstrate that a thin lanthanide-doped shell can function as an “energy relay” between the surface-anchored ICG sensitizers and the Er^3+^-rich core. Unlike an inert shell, this active interlayer shortens the effective energy transfer distance and enhances overall photon harvesting. To elucidate its mechanistic role, we systematically investigated a series of core-shell structures (NaErF_4_@NaYF_4_:x%Ln, Ln = Yb, Er, and Nd; denoted Er@xLn) and their ICG-sensitized counterparts. Our findings reveal that a Yb^3+^-rich active shell (NaYF_4_:50%Yb) enables an efficient cascaded energy-transfer pathway (ICG → Yb^3+^ → Er^3+^), dramatically enhancing 1525 nm emission. By contrast, an Er^3+^-doped shell only outperforms an inert shell beyond a critical thickness, as thinner Er-rich shells suffer from significant surface quenching that counteracts the sensitization benefit. The resulting optimal nanoprobe, Er@50Yb@ICG, demonstrates exceptional performance in deep-tissue NIR-IIb imaging, enabling high-contrast visualization of subcutaneous vasculature in mice. This work not only provides a high-performance imaging agent but also establishes Yb^3+^-mediated energy relay shells as a general design principle for engineering efficient energy transfer interfaces in dye-sensitized lanthanide nanomaterials.

## Results

**Fig. 1:**
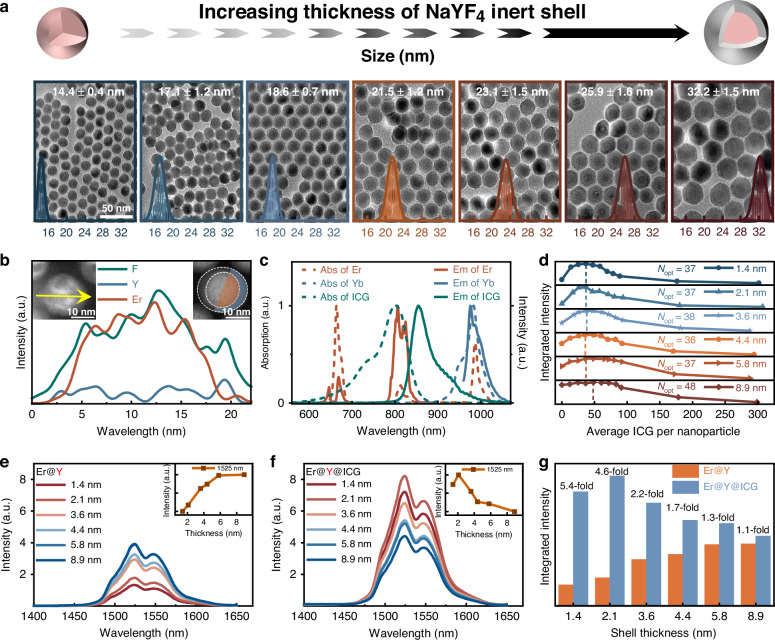
Structural and optical characterization of ICG-sensitized NaErF_4_@NaYF_4_. **a** Schematic illustration of increasing NaYF_4_ shell thickness, and TEM images of NaErF_4_@NaYF_4_-x nm with varying shell thickness (*x* = 0, 1.4, 2.1, 3.6, 4.4, 5.8, 8.9), scale bar: 50 nm. **b** Left: elemental line-scan profiles of a representative core-shell nanoparticle, showing spatial distributions of Er and Y along the yellow trace. Right: corresponding HAADF-STEM image of Er@Y-2.1 nm. **c** Absorption and emission spectra of Er^3+^, Yb^3+^, and ICG. **d** Integrated intensity versus dye concentration for Er@Y-x nm (x = 1.4, 2.1, 3.6, 4.4, 5.8, 8.9). (e) DSL spectra of unsensitized Er@Y under 808 nm excitation. Inset: integrated intensity as a function of inert shell thickness. **f** DSL spectra of ICG-sensitized Er@Y under 808 nm irradiation at optimal ICG concentration. Inset: integrated intensity as a function of inert shell thickness. **g** Quantitative comparison of integrated DSL intensities from (**e**, **f**). All DSL spectra were measured at a fixed irradiance of 7.0 W cm^−2^ at 808 nm

To establish a foundational understanding of distance-dependent energy transfer between ICG and Er^3+^ emitters, we first optimized the thickness of the inert NaYF_4_ shell. NaErF_4_ cores with an average diameter of 14.4 ± 0.4 nm (Fig. 1a) were synthesized via co-precipitation (see Materials and Methods for details)^40^. An inert NaYF_4_ shell was then epitaxially grown on these cores to suppress surface-related quenching. Given the strong dependence of dye-sensitization efficiency on the donor-acceptor distance, a series of Er@Y-x nm nanoparticles with precisely controlled shell thicknesses (*x* = 1.4, 2.1, 3.6, 4.4, 5.8, and 8.9) was prepared, as confirmed by transmission electron microscopy (TEM) images (Fig. 1a). High-angle annular dark-field scanning transmission electron microscopy (HAADF-STEM) images (Fig. 1b) show clear contrast between the core and shell regions, while elemental line-scan profiles across individual nanoparticles confirm the compositional transition from core to shell. X-ray diffraction (XRD) patterns (Figure S1) indicate a hexagonal phase in the nanoparticles, with no detectable phase change after NaYF_4_ shell growth.

ICG was selected as the sensitizing dye due to its strong spectral overlap with Er^3+^ absorption in the 800–850 nm range (Fig. 1c). Prior to dye conjugation, the native oleic acid ligands on the Er@Y surface were substituted with NOBF_4_. Notably, this ligand exchange did not induce measurable luminescence quenching upon transfer to DMF (Figure S2). ICG molecules were then anchored to the Er@Y surface via their sulfonic acid groups^41^ (Figure S3). Successful conjugation was verified by the characteristic ICG absorption band near 800 nm, and TEM images confirmed that the ICG-modified Er@Y nanoparticles remained well dispersed without aggregation (Figure S4).

The optimal dye-to-nanoparticle ratio for different shell thicknesses was determined by monitoring the 1525 nm downshifting luminescence (DSL) intensity (Fig. 1d). The emission intensity initially increased with dye concentration, reached saturation, and subsequently declined at higher concentrations. This decline is likely due to aggregation-caused quenching (ACQ) of the dye molecules^42,43^. For shell thicknesses below 5.8 nm, where FRET dominates sensitization, the optimal ratio was determined to be 37:1 (37 dye molecules per nanoparticle). By contrast, for the thickest shell (8.9 nm), the sensitization effect remained nearly constant ( ~ 1.1-fold) across a wide dye concentration range (25:1–69:1), reflecting the strong attenuation of FRET efficiency with increasing donor-acceptor distance (${\propto r}^{-6})$.

Next, the effect of shell thickness on the DSL performance at 1525 nm was examined. For consistent comparison, all spectra were normalized to the Er^3+^ absorption peak at 534 nm to ensure identical nanoparticle concentrations. As expected, unsensitized Er@Y samples under 808 nm excitation exhibited monotonically increasing emission with shell thickness (Fig. 1e), reflecting improved shielding of Er^3+^ from surface-related nonradiative decay pathways. By contrast, ICG-sensitized samples showed a non-monotonic dependence, with maximum enhancement (4.6-fold) at 2.1 nm (Fig. 1f). This trend reflects the interplay between surface quenching and donor-acceptor distance: shells thinner than 2.1 nm suffer from surface quenching despite efficient energy transfer, limiting net enhancement, while thicker shells ( > 2.1 nm) reduce FRET efficiency due to the increased distance between ICG and Er^3+^ core. Indeed, the energy transfer efficiency decreased from 27.7% (1.4 nm) to 1.7% (8.9 nm) (Figure S8), thereby diminishing the sensitization effect (Fig. 1g).

To further enhance 1525 nm DSL performance and probe the role of different shell dopants, we synthesized two series of core-shell nanoparticles with active shells: NaErF_4_@NaYF_4_:x%Er (Er@xEr; *x* = 0, 2, 5, 10, 20, 50) and NaErF_4_@NaYF_4_:x%Yb (Er@xYb; *x* = 0, 20, 50, 75, 100). To isolate the effect of shell dopants, the shell thickness ( ~ 2 nm) and the ICG-to-nanoparticle ratio (37:1) were meticulously maintained constant across all samples (Figures S9, S10). Emission spectra under 808 nm excitation were collected to directly correlate Er^3+^ or Yb^3+^ doping concentration with energy transfer dynamics and DSL performance.

**Fig. 2:**
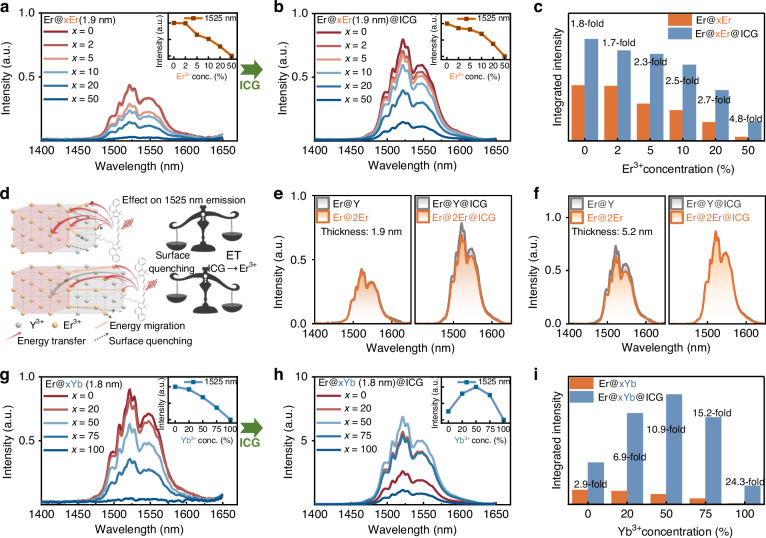
Effects of shell composition and thickness on the DSL of NaErF_4_-based nanoparticles. **a**, **b** DSL spectra of unsensitized and ICG-sensitized Er@xEr-1.9 nm series (*x* = 0, 2, 5, 10, 20, 50) under 808 nm excitation. **c** Quantitative comparison of integrated DSL intensities from (**a**, **b**). **d** Schematic illustration of the influence of active-shell thickness on 1525 nm emission. ET: energy transfer. **e**, **f** DSL spectra (with and without ICG) for Er@Y and Er@2Er nanoparticles with shell thicknesses of 1.9 nm and 5.2 nm, respectively. **g**, **h** DSL spectra of unsensitized and ICG-sensitized Er@xYb-1.8 nm series (*x* = 0, 20, 50, 75, 100) under 808 nm excitation. **i** Quantitative comparison of integrated DSL intensities from (**g**, **h**). All spectra were measured at a fixed irradiance of 7.0 W cm^−2^ at 808 nm

As shown in Fig. 2a, the 1525 nm DSL intensity decreases monotonically with increasing Er^3+^ doping concentration in the shell (0, 2, 5, 10, 20, and 50%). This decline indicates that surface quenching dominates at higher Er^3+^ concentrations, where rapid energy migration to surface defects becomes the primary nonradiative decay pathway for the ^4^I_11/2_ and ^4^I_13/2_ states^44^. Notably, even with optimal ICG concentration (Fig. 2b), which enhances the overall 1525 nm emission intensity, the decline persists, underscoring the overriding role of surface quenching over energy transfer. Interestingly, the enhancement factor rises with increasing Er^3+^ doping in the shell (Fig. 2c), revealing a competing interplay between two mechanisms: 1) positive contributions from ICG → Er^3+^ energy transfer, which improve with higher dopant density, and 2) negative impact from surface quenching, which escalates as more Er^3+^ populate near-surface regions. Thus, at lower doping levels, energy transfer dominates, yielding a net intensity gain; Beyond a critical concentration ( ~ 10% Er^3+^ in the shell), surface quenching prevails, as evidenced by the irreversible decline in 1525 nm emission, despite the rising enhancement factor. This interpretation is further supported by the comparative study of Er@2Er@ICG and Er@Y@ICG (Fig. 2d). Increasing the shell thickness to mitigate surface quenching allows the 1525 nm emission of Er@2Er@ICG to surpass that of Er@Y@ICG, underscoring the pivotal role of Er^3+^ in the active shell as a mediator by shortening ICG-Er^3+^ distances to boost energy transfer (Figs. 2e, f and S12). Ultimately, its efficiency is governed by the trade-off between enhanced sensitization and surface quenching—a balance tunable through dual optimization of shell thickness (to suppress quenching) and Er^3+^ concentration (to maximize proximal energy transfer).

**Fig. 3:**
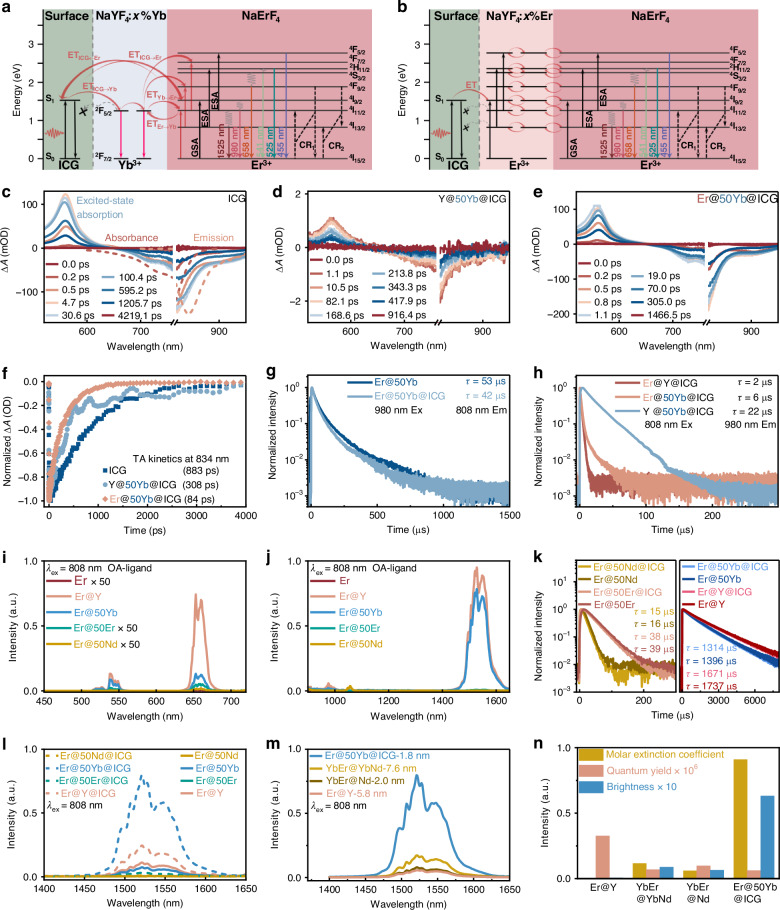
Investigation of energy transfer mechanisms and luminescence kinetics. Schematic of downshifting mechanism and the energy level diagrams of (**a**) Er@50Yb@ICG and (**b**) Er@50Er@ICG nanoparticles. Fs-TA spectra of (**c**) free ICG, (**d**) Y@50Yb@ICG, and (**e**) Er@50Yb@ICG. **f** Normalized TA kinetics probed at 834 nm for the corresponding samples. **g** Luminescence decay curves recorded at 808 nm emission under 980 nm pulsed laser excitation. **h** Luminescence decay curves recorded at 980 nm emission under 808 nm pulsed laser excitation. **i**, **j** Upconversion and downshifting luminescence spectra of NaErF_4_ core and core-shell nanoparticles (Er@Y, Er@50Yb, Er@50Er, and Er@50Nd). **k** Luminescence decay curves monitored at 1525 nm emission for the samples in (**j**). **l** DSL spectra comparing unsensitized and ICG-sensitized samples (Er@Y, Er@50Yb, Er@50Er, and Er@50Nd) under 808 nm excitation (7.0 W cm^−2^). **m** DSL spectra of Er@Y, YbEr@10Yb10Nd, YbEr@20Nd, and Er@50Yb@ICG) under 808 nm excitation (7.0 W cm^−2^). **n** Comparison of the molar extinction coefficient, quantum yield, and brightness of Er@Y, YbEr@10Yb10Nd, YbEr@20Nd, and Er@50Yb@ICG in DMF

Next, we selected Yb^3+^ as the active-shell dopant due to its efficient resonant energy transfer to Er^3+^ and larger absorption cross-section^43^. Similar to the Er@xEr system, the 1525 nm emission intensity of Er@xYb nanoparticles monotonically decreases with increasing Yb^3+^ concentration under both 808 nm (Fig. 2g) and 980 nm excitation (Figure S13), reflecting energy migration/transfer‑mediated surface quenching. Remarkably, upon ICG sensitization, the 1525 nm DSL intensity peaks at 50% Yb^3+^ (Fig. 2h), yielding nearly 10.9-fold enhancement (Fig. 2i). This result demonstrates that rationally designed energy cascades can overcome conventional concentration-quenching limits. To elucidate the distinct effects of the different active shells, we compared the energy level diagrams of Er@50Yb@ICG and Er@50Er@ICG under 808 nm excitation (Fig. 3a, b). Since Yb^3+^ in the interlayer cannot be directly excited at 808 nm, it serves as an energy reservoir that modulates energy flow via an ET_Er→Yb_ process, subsequently retransmitting energy back to Er^3+^. This cascade process markedly increases the population of the ^4^I_11/2_ level, thereby promoting the ^4^I_13/2_ → ^4^I_15/2_ transition of Er^3+^ at 1525 nm. To further verify this hypothesis, we established a simulation model incorporating these energy transfer processes (Figure S14). The results reveal that the Yb^3+^-mediated enhancement of the Er^3+ 4^I_13/2_ population is dictated by the interplay between ET_Er→Yb_ rate (ω) and ET_Yb→Er_ rate (ω_B_) energy transfer rates.

To gain deeper insights into the ICG-LnNPs energy-transfer dynamics, we performed femtosecond transient absorption (fs-TA) spectroscopy. Upon 800 nm excitation, free ICG (Fig. 3c) exhibits a positive excited-state absorption (ESA) band at 520-630 nm, along with two negative features corresponding to ground state bleaching and stimulated emission around 800 nm. These signals reach their maximum intensity within 4.7 ps and decay on the nanosecond timescale. By contrast, ICG integrated with LnNPs (Fig. 3d, e) shows that the ICG ESA signal peaks at 1.1 ps followed by ultrafast decay, indicating rapid energy transfer. Upon contacting different LnNPs, the excited-state lifetime of ICG decreases from 883 ps (free ICG) to 308 ps (Y@50Yb) and further to 84 ps (Er@50Yb) (Fig. 3f). This quenching corresponds to an energy transfer efficiency of ~90% (based on lifetime shortening), confirming that the Yb^3+^ sublattice serves as an efficient bridge for channeling excitation energy from ICG to Er^3+^.

Furthermore, to probe the directionality of energy flow, we conducted time-resolved spectroscopy. Under 980 nm excitation, which directly excites the Yb^3+^/Er^3+^ network, the shortened lifetime of the Er^3+ 4^I_9/2_ state (monitored at 808 nm) in Er@50Yb upon ICG coupling (Fig. 3g) confirms back energy transfer from Er^3+^ to ICG. Conversely, under 808 nm excitation, where ICG is primarily excited, the more rapid decay of the 980 nm emission in Er@50Yb@ICG compared with Y@50Yb@ICG (Fig. 3h) indicates that energy initially transferred from ICG to Yb^3+^ is subsequently funneled to Er^3+^. Collectively, these results demonstrate that the ICG → Yb^3+^ → Er^3+^ cascade enhances energy utilization efficiency, thereby boosting DSL.

To further elucidate the energy transfer mechanism in dye-sensitization, we investigated the impact of triplet quencher cyclooctatetraene (COT) on the DSL of Er@xYb@IR806, Er@xYb@ICG, and Er@xYb@disulfo-ICG (x = 0, 50) (Figure S15). Upon the addition of COT, the DSL intensity of Er@xYb@IR806 and Er@xYb@disulfo-ICG decreased significantly, indicating that the short-range triplet energy transfer is a critical factor in IR806-sensitized^45^ or disulfo-ICG-sensitized^41^ systems. Yb^3+^ enhances the heavy atom effect^46,47^, leading to an increased triplet state population in Er@50Yb@disulfo-ICG, which is strongly quenched by COT. By contrast, the DSL of Er@xYb@ICG is slightly enhanced upon COT addition, suggesting that ICG sensitization proceeds primarily via a singlet energy transfer pathway, bypassing triplet involvement.

Beyond acting as an energy bridge, the Yb^3+^-doped shell also protects Er^3+ 4^I_13/2_ level. Concentration quenching in Er^3+^-rich systems is primarily governed by two processes: cross-relaxation (CR) for high-energy states (^4^S_3/2_, ^4^F_9/2_, ^4^I_9/2_), and rapid energy migration to surface defects for low-energy states (^4^I_11/2_, ^4^I_13/2_)^44^. Spectral data (Fig. 3i, j) reveal distinct quenching trends for these levels. Specifically, the inert shell (Er@Y) maximizes emission by fully passivating surface quenching, whereas the 50% Yb^3+^-doped shell (Er@50Yb) presents a trade-off: energy transfer from Yb^3+^ to Er^3+^ (Yb^3+^: ^2^F_5/2_ → Er^3+^: ^4^I_11/2_ → ^4^I_13/2_) repopulates the ^4^I_13/2_ state, prolonging its decay lifetime (Fig. 3k), but concurrent Yb^3+^-defect coupling reduces 1525 nm intensity by 19%. The pronounced 84% drop in 650 nm emission underscores the role of Yb^3+^ in diverting ^4^I_11/2_ energy away from upconversion. By contrast, Er^3+^-/Nd^3+^-doped shells (Er@50Er and Er@50Nd) accelerate energy migration and surface coupling via resonant energy levels (^4^I_9/2_ for Er^3+^ and ^4^F_5/2_ for Nd^3+^), resulting in negligible 1525 nm emission.

Building on this, the ICG-sensitized Er@50Yb hybrid system exhibited dramatic enhancements in 1525 nm emission: 1965-fold relative to the core and 11-fold relative to its counterpart (Figs. 3l and S16). Benchmarking against classical Nd-sensitized systems (YbEr@20Nd and YbEr@10Yb10Nd^48^) and inert-shell Er@Y-5.8 nm revealed 10‑, 5‑, and 14‑fold higher DSL intensity, respectively (Fig. 3m). Quantification of absolute brightness, derived from quantum yield and molar extinction coefficient (Fig. 3n and Table S10), further confirmed its superior luminescence performance.

**Fig. 4:**
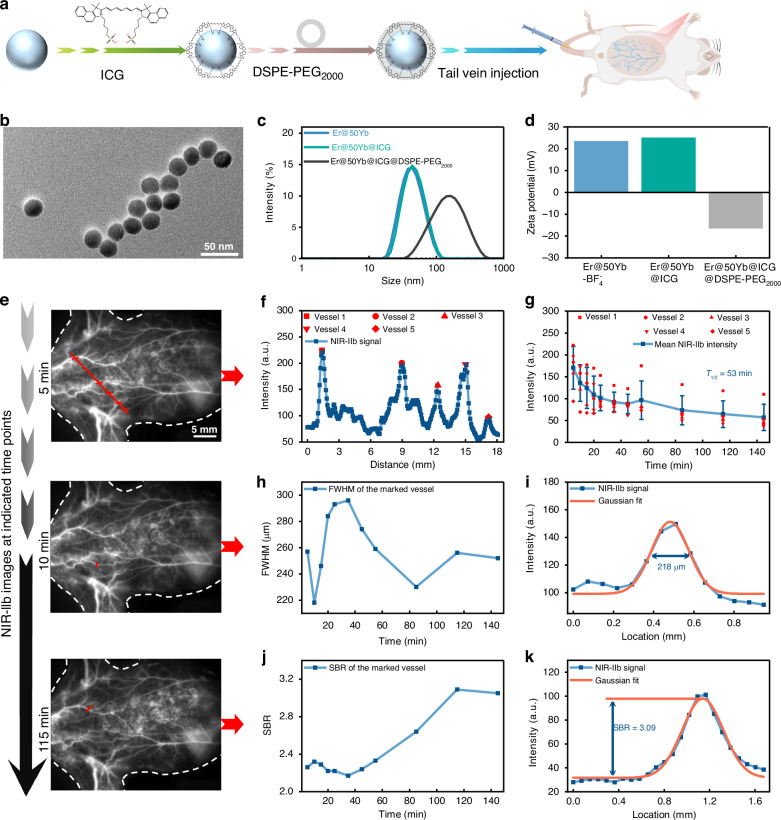
Characterization and in vivo evaluation of Er@50Yb@ICG@DSPE-PEG_2000_ nanoprobes. **a** Schematic illustration and (**b**) representative TEM image of the nanoprobes, scale bar: 50 nm. **c** Hydrodynamic size and (**d**) Zeta potential of Er@50Yb-BF_4_^−^, Er@50Yb@ICG, and Er@50Yb@ICG@DSPE-PEG_2000_ dispersed in deionized water. **e** Real-time in vivo NIR-IIb vascular imaging of mice following i.v. injection of nanoprobe under 808 nm irradiation (113 mW cm^−2^). **f** Quantification of luminescence intensity from the blood vessels at 5 min postinjection. **g** Blood circulation profile of nanoprobe over time (mean ± s.d., *n* = 5). **h** Full width at half maximum (FWHM) and (**j**) signal-to-background ratio (SBR) of the vessel indicated by red line in (**e**) over time. Luminescence intensity profiles across the vessel at (**i**) 10 min and (**k**) 115 min postinjection

Given its exceptional DSL brightness and suitable size, Er@50Yb@ICG was further PEGylated with DSPE-PEG_2000_ to improve hydrophilicity and biocompatibility for subsequent NIR-Ⅱb bioimaging studies (Fig. 4a). After PEGylation, the resulting Er@50Yb@ICG@DSPE-PEG_2000_ nanoprobe showed no obvious aggregation, as evidenced by the TEM image in Fig. 4b. Dynamic light scattering (DLS) revealed a hydrodynamic diameter of 133.2 nm (Fig. 4c) and a zeta potential of –16.5 mV (Fig. 4d), indicating high colloidal stability. No significant size change, dissociation, or precipitation was observed over 10 days in aqueous solution (Figure S17), confirming excellent dispersion stability. Under 808 nm irradiation at 330 mW cm^−^^2^ (three times the imaging power density), the luminescence intensity of the nanoprobe decreased by only ~20% after 60 min, demonstrating good photostability (Figure S18). Furthermore, 24 h MTT assays confirmed its low cytotoxicity, with cell viability exceeding 80% even at concentrations up to 800 μg mL^−1^ (Figure S19).

To evaluate imaging feasibility, we performed in vivo whole body vascular imaging and blood circulation monitoring in mice using Er@50Yb@ICG@DSPE-PEG_2000_. Luminescence detection at 1525 nm under 808 nm excitation provided deep-tissue penetration while minimizing overheating (Figure S20), leveraging the clarity of the NIR-IIb region. Our design strategically targets this 808/1525 nm excitation-emission pair is recognized as one of the most ideal combinations for NIR-IIb imaging. At 5 min postinjection via the tail vein, a strong NIR-IIb signal visualized the circulatory system, which gradually decreased but remained detectable for up to 2 h (Figs. 4e and S21). Vascular luminescence intensity along the red line at 5 min is shown in Fig. 4f. The nanoprobe exhibited a favorable blood half-time of 53 min (Fig. 4g), as quantified from the NIR-IIb signal decay measured across five vessels, enabling delineation of vascular hemodynamics under pathological conditions. Notably, the vessel width was determined to be 218 µm from the full width at half maximum (FWHM) of a Gaussian fit (Fig. 4i). Over time, the signal-to-background ratio (SBR) in murine blood vessels increased, peaking at 115 min, with a value of 3.09 (Fig. 4j, k). For vascular imaging in mice, the nanoprobe demonstrated excellent spatial resolution and high SBR (Table S9), enabling noninvasive visualization of vessel architecture and quantitative monitoring of hemodynamics. These properties establish Er@50Yb@ICG@DSPE-PEG_2000_ as a powerful tool for cardiovascular and oncological imaging in both physiological and pathological contexts.

## Discussion

In this work, we developed an ICG-sensitized sub-20 nm Er@50Yb nanoprobe with a thin Yb^3+^ active shell, which enables greatly enhanced 1525 nm DSL emission under 808 nm excitation. Compared to the bare NaErF_4_ core and inert-shell Er@Y counterpart, this approach yields 1965-fold and 11-fold enhancements, respectively, and outperforms classical Nd-sensitized systems (YbEr@20Nd, YbEr@10Yb10Nd) and inert-shell Er@Y-5.8 nm by 10-, 5-, and 14-fold. The resulting Er@50Yb@ICG architecture establishes the Yb^3+^ shell as an efficient energy-collection and relay layer that minimizes energy loss and promotes energy transfer to Er^3+^ emitters. The optimal 50% Yb^3+^ concentration balances energy relay efficiency against quenching. This cascaded mechanism effectively repopulates the ^4^I_13/2_ state, facilitating the Er^3+^: ^4^I_13/2_ → ^4^I_15/2_ transition at 1525 nm, yielding superior DSL performance from a structurally simple probe.

Following PEGylation, the probe exhibits excellent colloidal stability and biocompatibility. With its small size and exceptional brightness, it enables high-contrast in vivo vascular imaging in the NIR-IIb window with high spatial resolution. We note, however, that the intrinsic photobleaching of ICG remains a concern for long-term applications, and systematic evaluation of long-term toxicity and metabolic behavior is still needed. Overall, this study establishes an efficient and versatile strategy for engineering nanoscale contrast agents with strong potential for vascular imaging and broader biomedical applications.

## Materials and methods

### Chemicals and materials

Oleic acid (OA) ( > 90%) and 1-octadecene (ODE) ( > 90%) were purchased from Sigma-Aldrich. Indocyanine green (ICG) was supplied by Shanghai Yuanye Bio-Technology Co., Ltd. Disulfo-ICG was acquired from Confluore. ErCl_3_·6H_2_O (99.99%), YbCl_3_·6H_2_O (99.99%), and YCl_3_·6H_2_O (99.99%) were purchased from Aladdin (Shanghai). Cyclooctatetraene (COT) was produced by TCI. DMF was obtained from Shanghai Dahe Chemicals Co., Ltd. NH_4_F, NaOH, methanol, and ethanol were purchased from Sinopharm Chemical Reagent Co., Ltd., China. DSPE-mPEG_2000_ was purchased from MELOPEG. All chemical reagents were of analytical grade and were used directly without further purification.

### Synthesis of β-NaErF_4_ core

Firstly, 2 mmol ErCl_3_·6H_2_O was added to a 100 mL flask containing a mixture of oleic acid (20 mL) and 1-octadecene (20 mL). The mixture was stirred under vacuum and heated to 120°C, where it was maintained for 40 min to remove the water. After the solution became completely clear, it was cooled to 60°C.

Additionally, a methanolic solution (7 mL) containing NaOH (5 mmol) and NH_4_F (8 mmol) was added dropwise to the mixture. Under an argon atmosphere, the mixture was heated to 70°C and maintained at this temperature for 60 min to evaporate methanol. Next, the sample was heated to 300°C ( ~ 10°C/min) under argon protection and maintained for 1.5 h. The system was then cooled down to room temperature.

The as-prepared nanoparticles were precipitated by adding ethanol (40 mL) and collected by centrifugation (7500 rpm for 6 min). The supernatant was discarded. The products were washed twice with a 1:1 (v/v) cyclohexane/ethanol mixture (20 mL) and then stored in 8 mL of cyclohexane for subsequent shell coating.

### Synthesis of NaREF_4_ (RE: Y, Yb, and Er with various doping ratios) shell precursor

First, the rare earth oxides (RE_2_O_3_, RE=Y, Yb, and Er) were converted to their corresponding trifluoroacetate salts, (CF_3_COO)_3_RE, according to a previously reported method. Subsequently, CF_3_COONa (2 mmol) and the as-prepared (CF_3_COO)_3_RE mixture (2 mmol, calculated by a stoichiometric ratio) were placed in a 100 mL flask, followed by the addition of 20 mL OA and 20 mL ODE. The mixture was stirred under vacuum and heated to 120°C for 30 min to ensure complete dissolution of the reagents. It was then cooled to room temperature for further use.

### Synthesis of core-shell NaErF_4_@NaYF_4_

The core-shell NaErF_4_@NaYF_4_ was synthesized using a previously reported method^49^, initiated by adding 2 mL (0.5 mmol) of NaErF_4_ seed solution in cyclohexane. The resulting mixture was then heated to 120°C and maintained under vacuum for 30 min to remove low-boiling solvents, followed by a temperature increase to 300°C. Subsequently, 0.5 mmol of the α-NaYF_4_ shell precursor was injected into the three-necked flask using a syringe in four separate injections, each spaced at 25-minute intervals. Following the final injection, the mixture was heated again to 300°C and maintained for an additional 45 min to ensure a complete conversion of the shell precursor. Next, the solution was cooled to room temperature and subjected to two rounds of centrifugation with ethanol to isolate the final nanoparticles.

Similarly, NaErF_4_@NaYF_4_:x%Er, NaErF_4_@NaYF_4_:x%Yb, NaErF_4_@NaYF_4_:50%Nd, NaYF_4_@NaYF_4_:50%Yb, NaYF_4_:20%Yb,2%Er@NaYF_4_:20%Nd, and NaYF_4_:20%Yb,2%Er@NaYF_4_: 10%Yb,10%Nd nanoparticles were prepared.

### Ligand-exchange of nanoparticles and ICG titration procedures

To convert the OA-capped nanoparticles into hydrophilic, positively charged counterparts, a ligand exchange with NOBF_4_ was performed. Typically, 1 mL of the as-prepared nanoparticle dispersion in cyclohexane (0.125 mmol) was combined with 1 mL of a saturated NOBF_4_ solution in DMF. After gentle shaking for 3 min (until stratification), the nanoparticles were isolated by centrifugation (12,000 rpm for 30 min) and then redispersed in 1 mL of DMF.

### Preparation of dye-sensitized LnNPs

For dye sensitization, the prepared nanoparticles were mixed with varying volumes of dye (50 nM) in DMF. The mixture was gently shaken for 30 min to form ICG-LnNPs. Subsequently, the luminescence spectra were measured without centrifugation.

### PEGylation of NaErF_4_@NaYF_4_:50%Yb@ICG

The as-prepared NaErF_4_@NaYF_4_ nanoparticles in DMF (1 mL) were dispersed in chloroform (3 mL). The dispersion was mixed with a DMF solution of ICG (15 μL, 50 nM) and stirred for 2 min. Subsequently, the resulting Er@50Yb@ICG was mixed with a chloroform solution (3 mL) containing DSPE-PEG_2000_ (10 mg) in a 10 mL round-bottom flask. After stirring for 1 h at room temperature, the chloroform was removed by rotary evaporation. The resulting film was hydrated with 1 mL of deionized water. The hydrated suspension was transferred to a centrifugal tube, and excess lipids were discarded by low-speed centrifugation (1000 rpm, 5 min). The DSPE-PEG modified Er@50Yb@ICG were then purified via ultracentrifugation (12 000 rpm, 20 min). Finally, the precipitate was redispersed in 1.5 mL of deionized water.

### CCK8 assay

The cytotoxicity of the Er@50Yb@ICG@DSPE-PEG_2000_ probe was assessed using a standard CCK8 assay. Typically, L929 cells were seeded in a 96-well plate and allowed to adhere overnight. After the cells reached confluence, they were treated with different concentrations of probes and incubated for 24 h. Subsequently, CCK8 solution was added to each well, and cell viability (%) was measured by absorbance using a Bio-Rad microplate reader.

### NIR-IIb imaging in vivo

The Animal Use and Care Committee at Guangxi Normal University approved all experimental procedures, which were conducted in accordance with the National Institutes of Health guidelines. BALB/c mice (6–8 weeks old) were provided by Liaoning Changsheng Biotechnology Company for the in vivo study. After intravenously injecting Er@50Yb@ICG@DSPE-PEG_2000_, imaging was performed under 800 nm excitation ( ~ 113 mW cm^−2^). The mice were anaesthetized and placed on the imaging stage. In vivo NIR-II imaging was carried out using the InGaAs detector (SD 640, Tekwin, 50 ms high-gain model) with various filters (long-pass filters with a cut-on wavelength of 850 nm (FELH 850 nm, Thorlabs), 1000 nm (FELH 1000 nm, Thorlabs), and 1100 nm (FELH 1100 nm, Thorlabs) were used, respectively).

### Characterization

Transmission electron microscopy (TEM) was performed using a JEM 2100 F microscope operating at 200 kV. Scanning electron microscopy (SEM) was performed using a Hitachi S-4800 field-emission scanning electron microscope. Power X-ray diffraction (XRD) analysis was conducted using a Bruker D8-Advance diffractometer. The quantum yield (QY) and spectra were assessed using an Edinburgh FLS 980 spectrophotometer, employing excitation sources at 980 and 808 nm. Absorption spectra were recorded with an Ocean Optics Maya-2000 fiber optic spectrometer. Fluorescence lifetimes were measured using a Hamamatsu R9110 PMT single-photon counting system. The luminescence lifetime was analyzed by fitting the decay profile to a single-exponential model ($y={y}_{0}+A\times {e}^{-x/t}$). Particle size was determined by dynamic light scattering using a Malvern Zetasizer Nano.

## Supplementary information

Supplementary Information for Dye-Sensitized Cascaded Energy Transfer Amplification of 1525 nm Luminescence in High-Doped Nanoparticles

## Data Availability

All data generated or analyzed during this study are included in the published article and its Supplementary Information. Additional data are available from the corresponding author upon request.
