# Supplementary material for: Dye-sensitized cascaded energy transfer for amplified 1525 nm luminescence in highly doped lanthanide nanoparticles
Source: Light Sci Appl. 2026 Apr 27;15:215. doi: 10.1038/s41377-026-02302-9 (PMC13121758; doi:10.1038/s41377-026-02302-9)
Supplement: Supplementary file 1 — Supplementary Information for Dye-Sensitized Cascaded Energy Transfer Amplification of 1525 nm Luminescence in High-Doped Nanoparticles [file 41377_2026_2302_MOESM1_ESM.pdf]

Supplementary Information for

Dye-Sensitized Cascaded Energy Transfer for Amplified 1525 nm Luminescence in Highly Doped Lanthanide Nanoparticles

Fei Long[1,2], Dechao Gan[1,2], Haoran Chen[1], Qiqing Li[3], Wang Wang[1], Zexuan Sui[1], Youlin Zhang[4], Dabing Li[1], and Yulei Chang[1,]*

[1]*State Key Laboratory of Luminescence Science and Technology, Changchun Institute of Optics, Fine Mechanics and Physics, Chinese Academy of Sciences, Changchun 130033, China;*

[2]*University of the Chinese Academy of Sciences, Beijing 100049, China;*

[3]*College of Physics and Information Engineering, Fuzhou University, Fuzhou 350108, China;*

[4]*School of Chemistry and Pharmaceutical Sciences, Guangxi Normal University, Guilin 541000, China.*

**Correspondence: Yulei Chang (yuleichang@ciomp.ac.cn)*

Section 1. Tables 4

Table S1. Nanoparticles used in Figure 1. 4
Table S2. Nanoparticles used in Figure 2a-2c. 4
Table S3. Nanoparticles used in Figure 2e-2f. 4
Table S4. Nanoparticles used in Figure 2g-2i. 4
Table S5. Nanoparticles used in Figure 3c-3h. 5
Table S6. Nanoparticles used in Figure 3i-3l. 5
Table S7. Nanoparticles used in Figure 3m. 5
Table S8. Surface density of dye molecules for nanoparticle samples of different sizes. 5
Table S9. Statistical Table for SBR and FWHM of NIR-II Nanoprobes. 6
Table S10. Statistical Table of Quantum Yield (***Φ***) and Absolute Brightness (***B***). 6

Section 2. Formula 7

Formula S1. Calculation of dye-to-nanoparticle ratio. 7
Formula S2. Calculation formula of spectral overlap integral. 8
Formula S3. Lifetime Fitting Formula. 8
Formula S4. Calculation of absolute brightness values. 8

Section 3. Supplemental Data 9

Figure S1. XRD patterns of the core $NaErF_4$ and a representative core-shell sample. 9
Figure S2. Emission spectra of Er@Y nanoparticles in cyclohexane and in DMF. 10
Figure S3. FTIR spectra of Er@Y before and after $NOBF_4$ treatment, and of Er@Y@ICG. 10
Figure S4. The characterization of Er@Y before and after dye modification. 11
Figure S5-S6. The absorption and emission spectra of Er@Y connected to different amounts of ICG. 11
Figure S7. Determination of ICG, Er^{3+}, Yb^{3+}, and Nd^{3+} concentration. 13
Figure S8. Energy transfer efficiency from ICG to Er^{3+} in samples with different shell thicknesses. 14
Figure S9. TEM Characterization. 15
Figure S10. Absorption spectra of Er@xEr@ICG (x = 0, 2, 5, 10, 20, 50%) and Er@xYb@ICG (x = 0, 20, 50, 75, 100%). 16
Figure S11. Emission spectra of unsensitized and ICG-sensitized Er@xEr-5.2 nm (x = 0, 2, 5, 10, 20, 50%). 16
Figure S12. DSL spectra (with and without ICG) for Er@Y and Er@2Er nanoparticles with different shell thicknesses. 17
Figure S13. Emission spectra of unsensitized and ICG-sensitized Er@xYb-1.8 nm (x = 0, 20, 50, 75, 100%) under 980 nm excitation. 17
Figure S14. Theoretical Model for Downshifting Luminescence and Energy Transfer

From Yb^{3+} to Er^{3+} ($ET_{Yb \rightarrow Er}$). 18
Figure S15. Investigation of the Energy Transfer Mechanism via Triplet State Quenching. 20
Figure S16. Repeatability testing. 21
Figure S17. Stability of Er@50Yb@ICG@DSPE-PEG$_{2000}$ monitored by DLS. 21
Figure S18. The photostability of Er@50Yb@ICG@DSPE-PEG$_{2000}$. 21
Figure S19. CCK8 Assay. 22
Figure S20. Experiment on the Penetration Depth of NIR Lights. 23
Figure S21. In vivo NIR-IIb imaging. 24

Section 4. References 25

Section 1. Tables

Table S1. Nanoparticles used in Figure 1.

Structure composition	Shell thickness	Abbreviation	Enhancement factor*
$NaErF_4@NaYF_4$	1.4 nm	Er@Y-1.4 nm	5.4
$NaErF_4@NaYF_4$	2.1 nm	Er@Y-2.1 nm	4.6
$NaErF_4@NaYF_4$	3.6 nm	Er@Y-3.6 nm	2.2
$NaErF_4@NaYF_4$	4.4 nm	Er@Y-4.4 nm	1.7
$NaErF_4@NaYF_4$	5.8 nm	Er@Y-5.8 nm	1.3
$NaErF_4@NaYF_4$	8.9 nm	Er@Y-8.9 nm	1.1

* The enhancement factor refers to the fold increase in the 1525 nm emission of the sample after ICG sensitization relative to its own emission before sensitization.

Table S2. Nanoparticles used in Figure 2a-2c.

Structure composition	Abbreviation	Enhancement factor*
$NaErF_4@NaYF_4$	Er@Y-1.9 nm	1.8
$NaErF_4@NaYF_4$: 2%Er	Er@2Er-1.9 nm	1.7
$NaErF_4@NaYF_4$: 5%Er	Er@5Er	2.3
$NaErF_4@NaYF_4$: 10%Er	Er@10Er	2.5
$NaErF_4@NaYF_4$: 20%Er	Er@20Er	2.7
$NaErF_4@NaYF_4$: 50%Er	Er@50Er	4.8

Table S3. Nanoparticles used in Figure 2e-2f.

Structure composition	Abbreviation	Enhancement factor*
$NaErF_4@NaYF_4$	Er@Y-1.9 nm	1.8
$NaErF_4@NaYF_4$: 2%Er	Er@2Er-1.9 nm	1.7
$NaErF_4@NaYF_4$	Er@Y-5.2 nm	1.2
$NaErF_4@NaYF_4$: 2%Er	Er@2Er-5.2 nm	1.4

Table S4. Nanoparticles used in Figure 2g-2i.

Structure composition	Abbreviation	Enhancement factor*
$NaErF_4@NaYF_4$	Er@Y-1.8 nm	2.9
$NaErF_4@NaYF_4$: 20%Yb	Er@20Yb	6.9

$NaErF_4@NaYF_4$: 50%Yb	Er@50Yb	10.9
$NaErF_4@NaYF_4$: 75%Yb	Er@75Yb	15.2
$NaErF_4@NaYF_4$: 100%Yb	Er@100Yb	24.3

Table S5. Nanoparticles used in Figure 3c-3h.

Structure composition	Abbreviation	Enhancement factor*
Indocyanine green	ICG	None
$NaYF_4@NaYF_4$: 50%Yb	Y@50Yb	None
$NaErF_4@NaYF_4$: 50%Yb	Er@50Yb	None
$NaErF_4@NaYF_4$	Er@Y	None

Table S6. Nanoparticles used in Figure 3i-3l.

Structure composition	Abbreviation	Relative enhancement factor to Er-core**
$NaErF_4$	Er-core	None
$NaErF_4@NaYF_4$	Er@50Y	575
$NaErF_4@NaYF_4$: 50%Yb	Er@50Yb	1965 ± 55
$NaErF_4@NaYF_4$: 50%Er	Er@50Yb	73
$NaErF_4@NaYF_4$: 50%Nd	Er@50Nd	20

**The relative enhancement factor relative to Er-core refers to the luminescence enhancement factor of this sample after ICG sensitization relative to $NaErF_4$.

Table S7. Nanoparticles used in Figure 3m.

Structure composition	Abbreviation	Enhancment factor***
$NaErF_4@NaYF_4$:50%Yb@ICG	Er@50Yb@ICG-1.8 nm	1
$NaYF_4$:20%Yb,2%Er@$NaYF_4$:20%Nd	YbEr@Nd-2 nm	10
$NaYF_4$:20%Yb,2%Er@$NaYF_4$:10%Yb,10%Nd	YbEr@YbNd-7.6 nm	5
$NaErF_4@NaYF_4$	Er@Y-5.8 nm	14

***Enhancement factor of the luminescence intensity of Er@50Yb@ICG relative to that of the sample.

Table S8. Surface density of dye molecules for nanoparticle samples of different sizes.

Sample	Surface density (molecules nm^{-2})
Er@Y-1.4 nm	1.4×10^{-2}

Er@Y-2.1 nm	1.1×10^{-2}
Er@Y-3.6 nm	7.1×10^{-3}
Er@Y-4.4 nm	5.7×10^{-3}
Er@Y-5.8 nm	4.1×10^{-3}
Er@Y-8.9 nm	2.8×10^{-3}

Table S9. Statistical Table for SBR and FWHM of NIR-II Nanoprobes.

Structure composition	Excitation wavelength	Imaging wave-length	Imaging area	SBR	FWHM	Refs
Tm(02Er)-NPs and Er-NPs (of identical concen-trations)	808 nm (100 mW cm^{-2})	1500-1700 nm	blood vessels	1.27	None	1
		≥1700 nm		1.56		
$NaYbF_4$: 5 %Tm@$NaYbF_4$ @$NaYF_4$	980 nm (45 mW cm^{-2})	1852 nm	blood vessels	2.72	240 µm	2
	980 nm (58 mW cm^{-2})	1532 nm	lymph	5.92	780 µm	
$NaErF_4$: 2%Ce@$NaYbF_4$ @$NaYF_4$	980 nm (12.7 mW cm^{-2})	1525 nm	blood vessels	1.45	594.8 µm	3
$NaYF_4$@$NaYbF_4$: 2%Er,20%Ce @$NaYF_4$:15%Ca	975 nm	1550 nm	capillary vessels	2.75	77.62 µm	4
			a capillary vessel through the skull	8.82	15.21 µm	
$NaErF_4$@$NaYF_4$: 50%Yb@ICG	808 nm (113 mW cm^{-2})	1525 nm	blood vessels	3.09	218 µm	This work

Table S10. Statistical Table of Quantum Yield (***Φ***) and Absolute Brightness (***B***).

Sample	Molar extinction coefficient (ε) (L mol^{-1} cm^{-1})	Quantum yield (Φ)	Brightness (B) (L mol^{-1} cm^{-1})
Er@Y-5.8 nm	2068	35.89%	742
YbEr@YbNd-7.6 nm	127860	7.52%	9615

YbEr@Nd-2 nm	66481	10.74%	7140
Er@50Yb@ICG-1.8 nm	1001068	6.94%	69474

Section 2. Formula

Formula S1. Calculation of dye-to-nanoparticle ratio.

In the absorption spectra measurement, the concentration of Er^{3+} is 0.0014 mmol, and the samples are dispersed in 1 mL of DMF.

N of Er^{3+} in each sample= $n \times \mathrm{N_A} = 1.4 \times 10^{-6} \times 6.02 \times 10^{23} = 8.428 \times 10^{17}$

The calculation of the number of particles in Er@Y-x nm:

Size (***d***) of the core of Er@Y-x nm is found to be 14.4 nm (from TEM images)

Radius (***r***) of single nanoparticle (NP)= $\frac{d}{2} = \frac{14.4}{2}\, nm = 7.2\, nm$

Volume of single NP= $\frac{4}{3}\pi r^3 = \frac{4}{3} \times \pi \times 7.2^3 \approx 1563.46\, nm^3$

For the core $NaErF_4$[5], the cell parameter is $a = 5.959$ Å$, c = 3.487$ Å

Volume of a single unit cell $= \frac{\sqrt{3}}{2} a^2 c = \frac{\sqrt{3}}{2} \times 5.959^2 \times 3.487 = 107.23$ Å^3

Number of unit cells per NP $= \frac{1563.46\, nm^3}{107.23\, Å^3} = 1.46 \times 10^4$

Number of Er^{3+} in unit cell per NP= $1.46 \times 10^4 \times 1.5 = 2.19 \times 10^4$

Number of NPs= $\frac{N\ of\ Er^{3+}\ in\ each\ sample}{Number\ of\ Er^{3+}\ in\ unit\ cell\ per\ NP} = \frac{8.428 \times 10^{17}}{2.19 \times 10^4} = 3.85 \times 10^{13}$

In Figure 1d, according to Figure S5a, the optimal ***n*** of ICG is 2.39 nmol.

N of ICG= $n \times N_A = 2.39 \times 10^{-9} \times 6.02 \times 10^{23} \approx 1.44 \times 10^{15}$

Ratio of ICG to the nanoparticles =$\frac{N\ of\ ICG}{Number\ of\ NPs} = \frac{1.44 \times 10^{15}}{3.85 \times 10^{13}} = 37.40 \approx 37$

Formula S2. Calculation formula of spectral overlap integral.

The overlap integral ***J(λ)*** represents the degree of overlap between donor emission and acceptor absorption. The overlapping integral is given by the following Equation (1):

$$J(\lambda) = \int_0^\infty f_D(\lambda)\varepsilon_A(\lambda)\lambda^4 dx \tag{1}$$

$\boldsymbol{f_D}$ is the normalized emission intensity of the donor, $\boldsymbol{\varepsilon_A}$ refers to the molar absorption coefficient of the acceptor, and $\boldsymbol{\lambda}$ is the wavelength.

Formula S3. Lifetime Fitting Formula.

Almost all the luminescence decay curves of the materials in the article were fitted using Equation (2).

$$y = y_0 + A_1 e^{-\frac{x}{t_1}} \tag{2}$$

For the luminescence decay curves exhibiting dual decay processes at specific wavelengths mentioned in the manuscript, Equation (3) is used for fitting. The material lifetime was calculated using Equation (4).

$$y = y_0 + A_1 e^{-\frac{x}{t_1}} + A_2 e^{-\frac{x}{t_2}} \tag{3}$$

$$y = \frac{A_1 t_1^2 + A_2 t_2^2}{A_1 t_1 + A_2 t_2} \tag{4}$$

Formula S4. Calculation of absolute brightness values.

In the chemical sciences, it is expressed based on molar extinction coefficient (*ε*) and quantum yield ***(QY)*** of an emitter:

$$\boldsymbol{Brightness} = \boldsymbol{\varepsilon} \times \boldsymbol{QY} \quad (5)$$

typically in units of M^{-1} cm^{-1}.

In case of nanomaterials composed of multiple emitters (dyes), their brightness (***B***) depends on the number of emitters per NP, as follows[6]:

$$\boldsymbol{Brightness}\ (\boldsymbol{B}) = \boldsymbol{N} \times \boldsymbol{\varepsilon_{dye}} \times \boldsymbol{QY} \quad (6)$$

Where $\boldsymbol{\varepsilon_{dye}}$ is the extinction coefficient of one dye (emitter), ***N*** is the number of emitters per NP, and ***QY*** the quantum yield of the nanomaterial.

We measured the absorbance of each LnNPs at known concentrations at the excitation wavelength and calculated the molar extinction coefficient (ε) according to the Beer-Lambert law:

$$\boldsymbol{A} = \boldsymbol{\varepsilon} \times \boldsymbol{c} \times \boldsymbol{l} \quad (7)$$

A is the absorbance, also known as Optical Density (OD). ***ε*** is the molar attenuation coefficient, with units typically expressed as L mol^{-1} cm^{-1}. ***l*** is the path length, with units usually in cm. ***c*** is the molar concentration of the absorbing substance, with units typically in mol L^{-1}.

Section 3. Supplemental Data

Figure S1. XRD patterns of the core $NaErF_4$ and a representative core-shell sample.

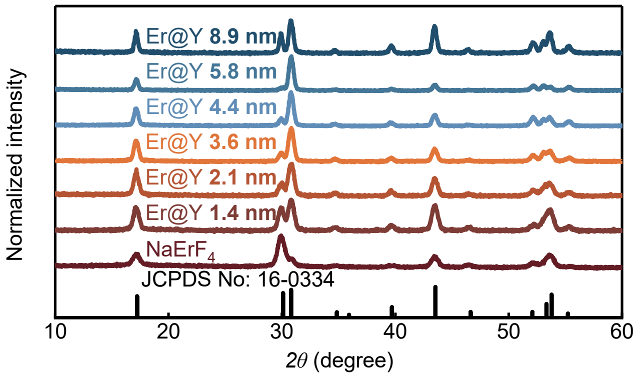

Figure S1. XRD patterns of the core $NaErF_4$ and a representative core-shell sample.

Figure S2. Emission spectra of Er@Y nanoparticles in cyclohexane and in DMF.

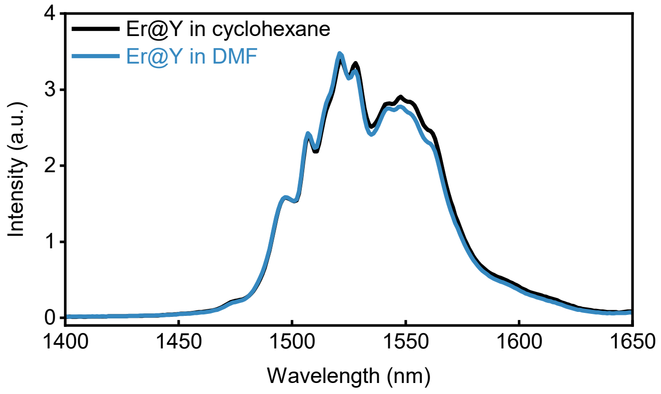

Figure S2. Comparison of the emission spectra of Er@Y nanoparticles dispersed in cyclohexane (as-synthesized, OA-capped) and in DMF (following ligand exchange with $NOBF_4$).

Figure S3. FTIR spectra of Er@Y before and after $NOBF_4$ treatment, and of Er@Y@ICG.

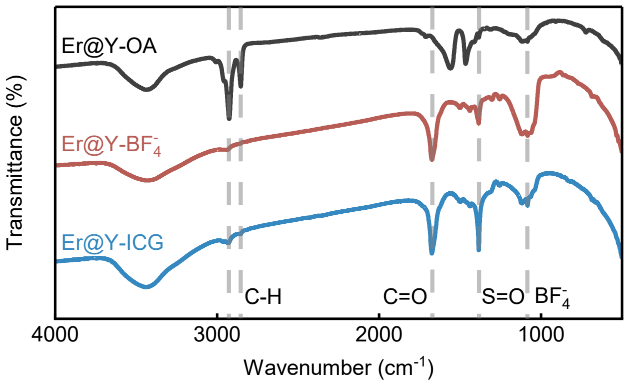

Figure S3. FTIR spectra of Er@Y before and after ligand exchange with $NOBF_4$ and of Er@Y@ICG. The characteristic peaks at 2932 and 2844 cm^{-1}, corresponding to the asymmetric and symmetric C-H stretching vibrations of the CH_2 group, were significantly attenuated after treatment with $NOBF_4$. A new peak emerged around 1080 cm^{-1}, which is assigned to the B-F stretching vibration of BF_4^- anions. These observations confirm the successful exchange of OA ligands with inorganic BF_4^- anions. The peak around 1680 cm^{-1} is attributed to the C=O stretching vibration from residual DMF molecules. After conjugation with ICG molecules, the peaks at 1382 cm^{-1} correspond to the sulfonic acid group present in ICG, confirming a

successful attachment of ICG to Er@Y surface.

Figure S4. The characterization of Er@Y before and after dye modification.

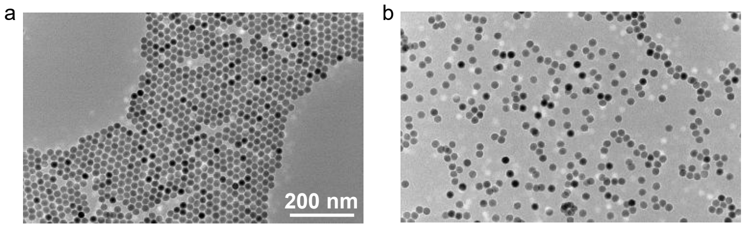

Figure S4. TEM images of a) Er@Y-4.4 nm and b) Er@Y@ICG-4.4 nm, scale bar: 200 nm.

Figure S5-S6. The absorption and emission spectra of Er@Y connected to different amounts of ICG.

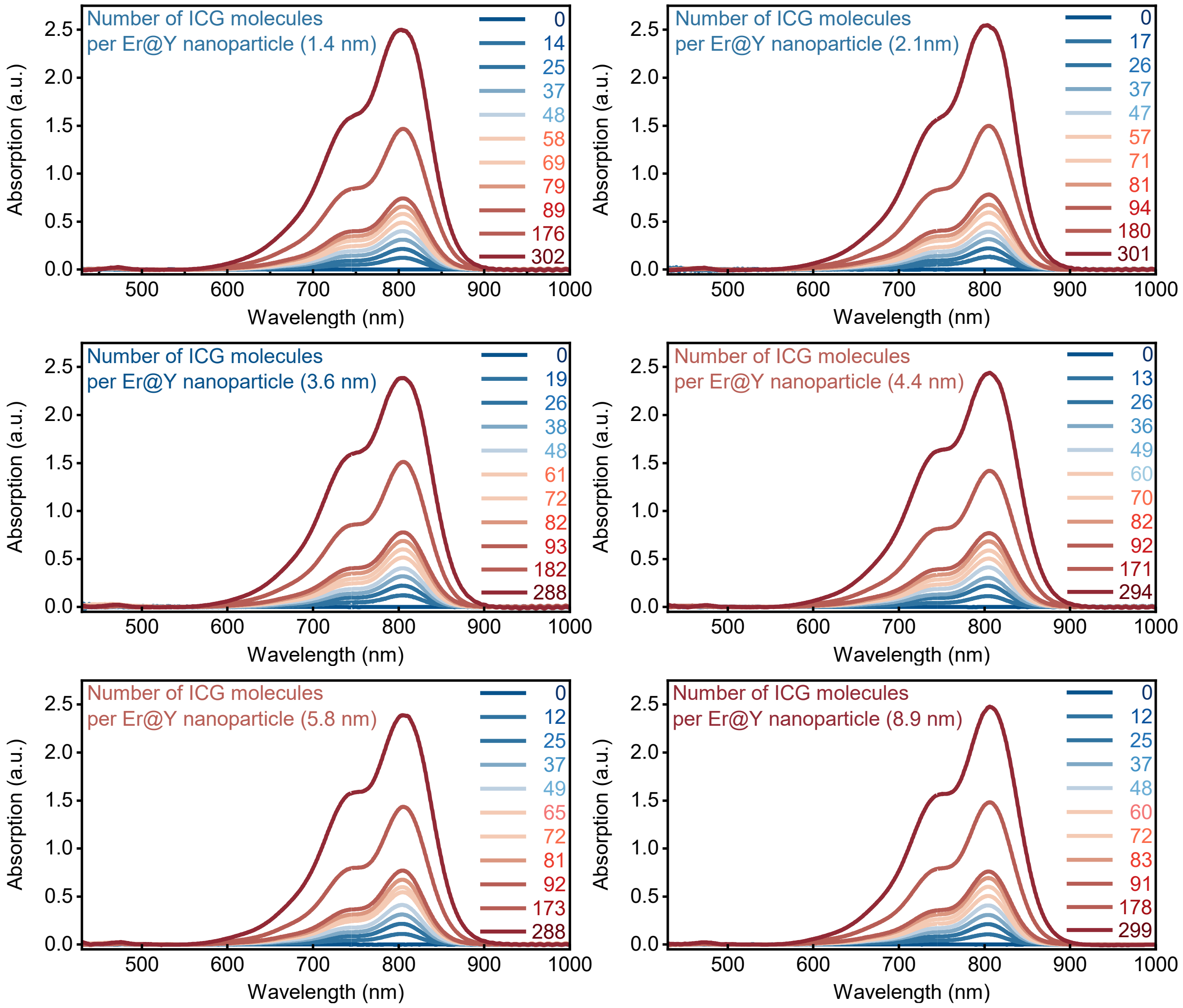

Figure S5. Absorption spectra of Er@Y-x nm (x = 1.4, 2.1, 3.6, 4.4, 5.8 and 8.9) conjugated to different amounts of ICG.

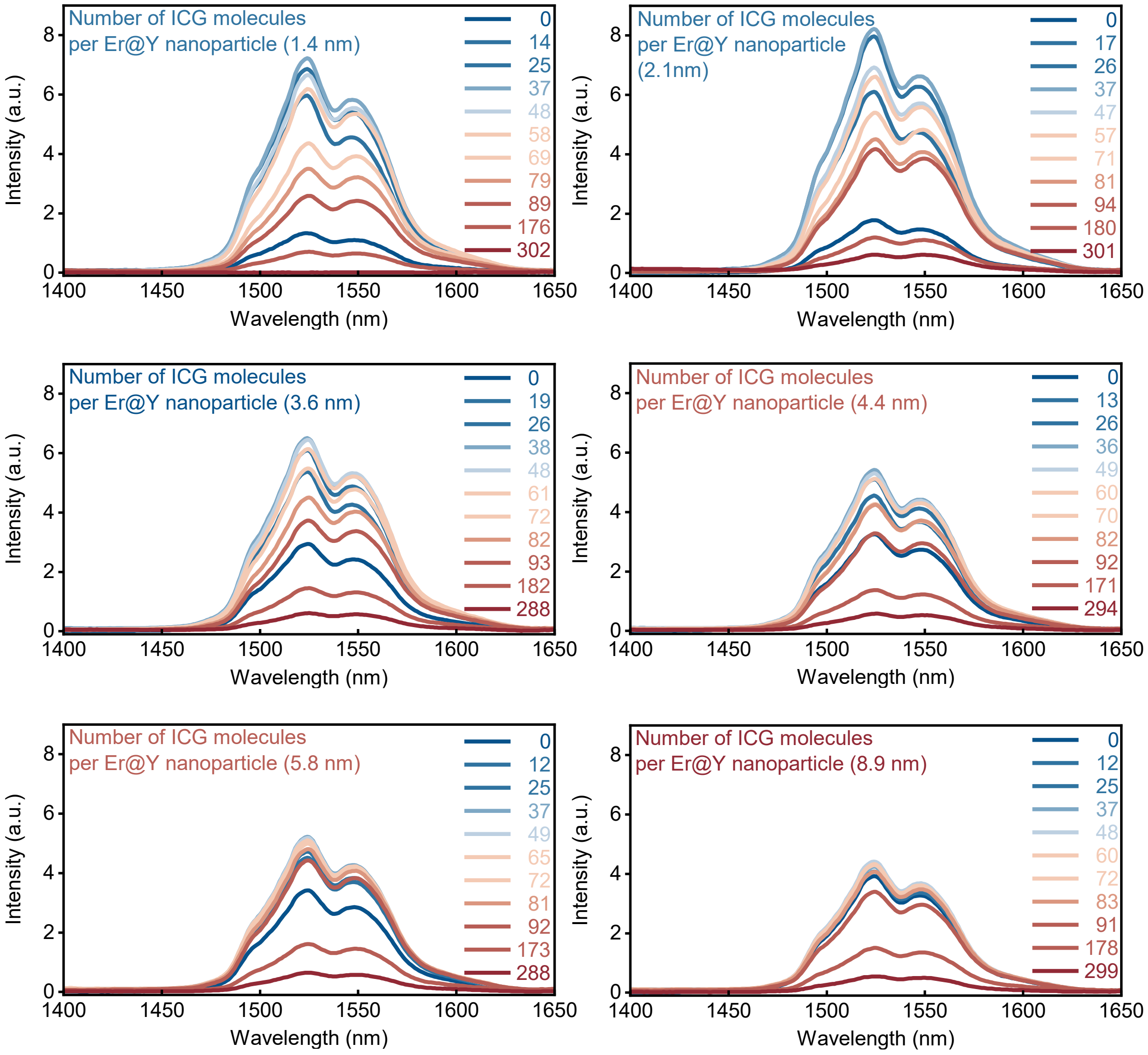

Figure S6. Emission spectra of Er@Y-x nm (x = 1.4, 2.1, 3.6, 4.4, 5.8 and 8.9) conjugated to different amounts of ICG under 808 nm excitation.

Figure S7. Determination of ICG, Er^{3+}, Yb^{3+}, and Nd^{3+} concentration.

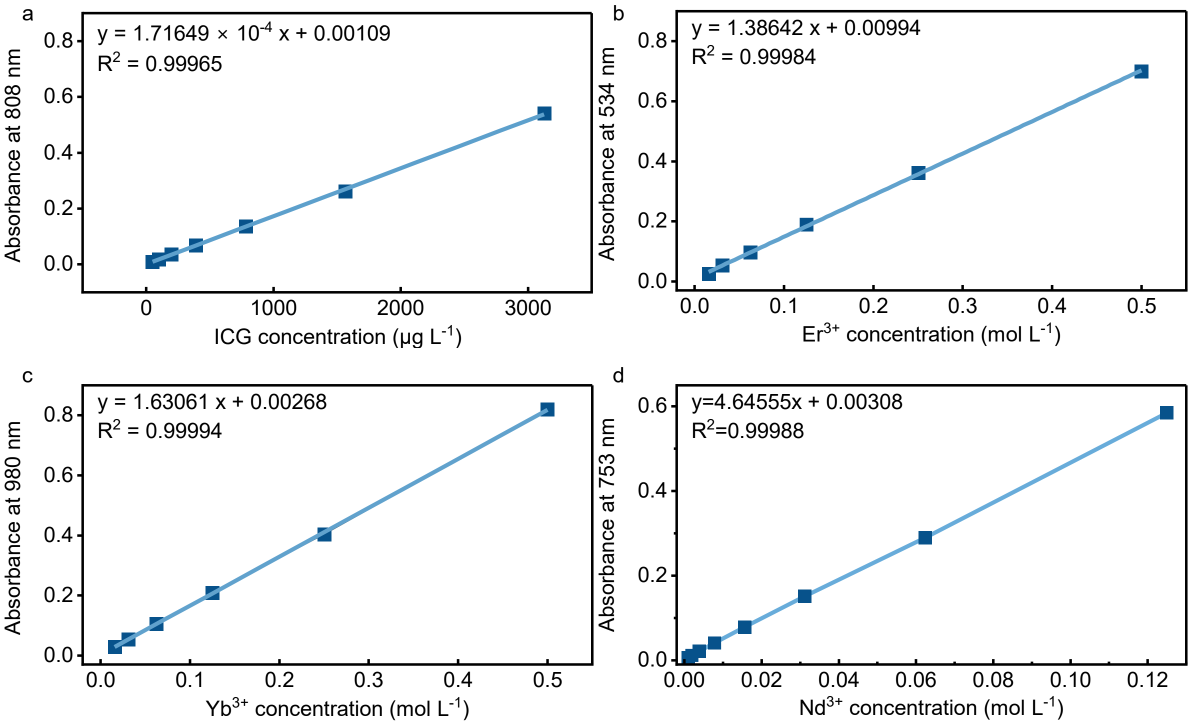

Figure S7. (a) Calibration curve of (a) ICG, (b) Er^{3+}, (c) Yb^{3+}, and (d) Nd^{3+} absorbance versus concentration.

(a) The ICG loading concentration on the nanoparticles was determined by measuring the absorption at 808 nm and comparing it to a standard ICG calibration curve.

(b) The absorption of Er-NPs at 534 nm, Yb-NPs at 980 nm, and Nd-NPs at 753 nm was measured. The concentrations of Er^{3+}, Yb^{3+}, or Nd^{3+} in the nanoparticles were calculated by comparing the measured absorbance to a calibration curve.

Figure S8. Energy transfer efficiency from ICG to Er^{3+} in samples with different shell thicknesses.

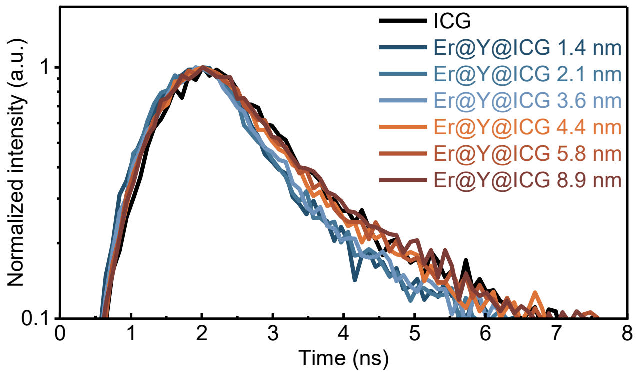

Figure S8. Time-resolved fluorescence decay curves of ICG used for energy transfer efficiency calculation.

Decay curves were monitored at 834 nm under 635 nm pulsed excitation.

The energy transfer efficiency from ICG to Er^{3+} can be calculated as:

$$\eta_{ET} = 1 - \frac{\tau_{Er@Y@ICG}}{\tau_{ICG}}$$

Sample	Lifetime (ns)	η_{ET}
ICG	1.66	None
Er@Y@ICG-1.4 nm	1.20	27.7%
Er@Y@ICG-2.1 nm	1.27	23.3%
Er@Y@ICG-3.6 nm	1.39	16.2%
Er@Y@ICG-4.4 nm	1.55	6.8%
Er@Y@ICG-5.8 nm	1.58	4.9%
Er@Y@ICG-8.9 nm	1.63	1.7%

Figure S9. TEM Characterization.

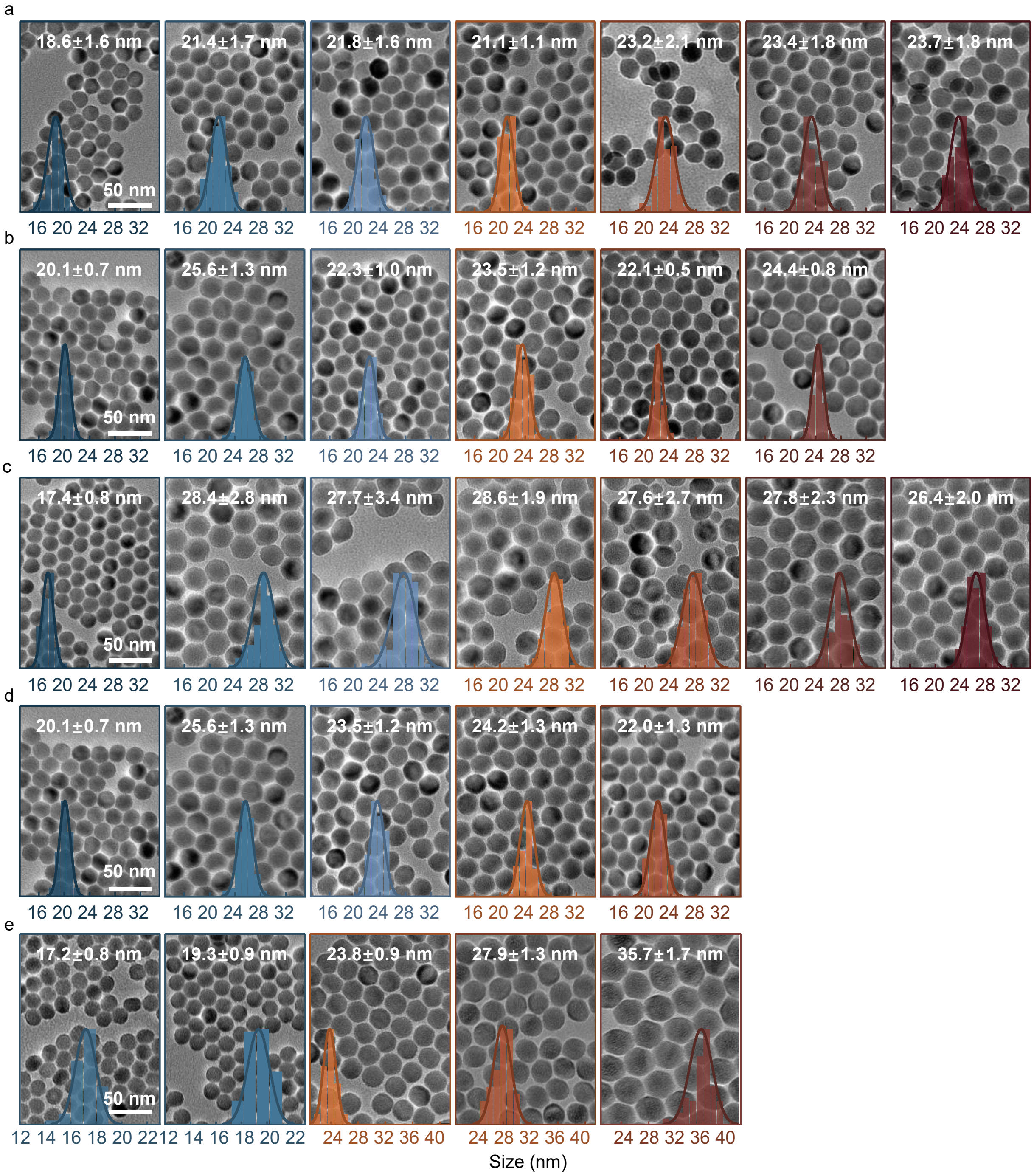

Figure S9. (a) TEM images of $NaErF_4$ and Er@xEr-1.9 nm with varying Er^{3+} concentration (x = 0, 2, 5, 10, 20, 50%), scale bar: 50 nm. (b) TEM images of $NaErF_4$ and Er@xYb-1.8 nm with varying Yb^{3+} concentration (x = 0, 20, 50, 75, 100%), scale bar: 50 nm. (c) TEM images of $NaErF_4$ and Er@xEr-5.2 nm with varying Er^{3+} concentration (x = 0, 2, 5, 10, 20, 50%), scale bar: 50 nm. (d) TEM images of $NaErF_4$, Er@Y, Er@50Yb, Er@50Er and Er@50Nd, scale bar: 50 nm. (e) TEM images of $NaYF_4$, Y@50Yb, $NaYF_4$: 20%Yb,2%Er,

YbEr@Nd and YbEr@YbNd, scale bar: 50 nm.

Figure S10. Absorption spectra of Er@xEr@ICG (x = 0, 2, 5, 10, 20, 50%) and Er@xYb@ICG (x = 0, 20, 50, 75, 100%).

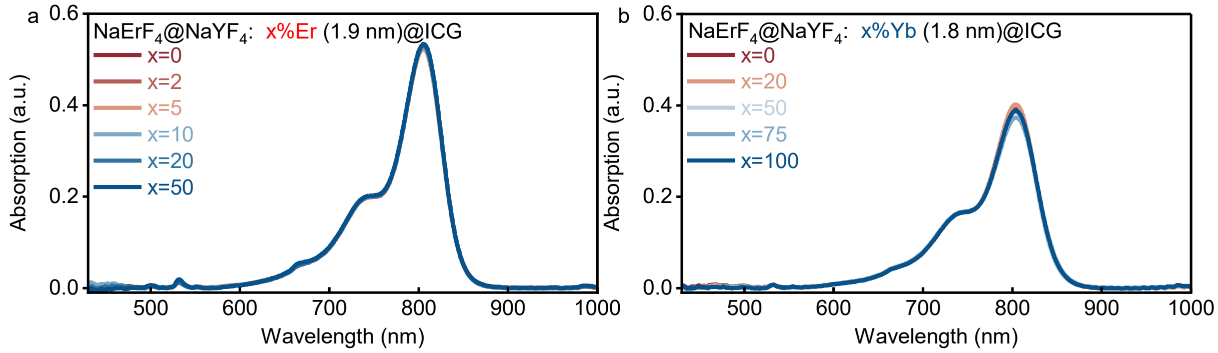

Figure S10. (a) Absorption spectra of Er@xEr-1.9 nm@ICG with varying Er^{3+} concentrations (x = 0, 2, 5, 10, 20, 50%). (b) Absorption spectra of Er@xYb-1.8 nm@ICG with varying Yb^{3+} concentrations (x = 0, 20, 50, 75, 100%).

The spectral consistency indicates a constant dye-to-nanoparticle ratio of approximately 37 for both series.

Figure S11. Emission spectra of unsensitized and ICG-sensitized Er@xEr-5.2 nm (x = 0, 2, 5, 10, 20, 50%).

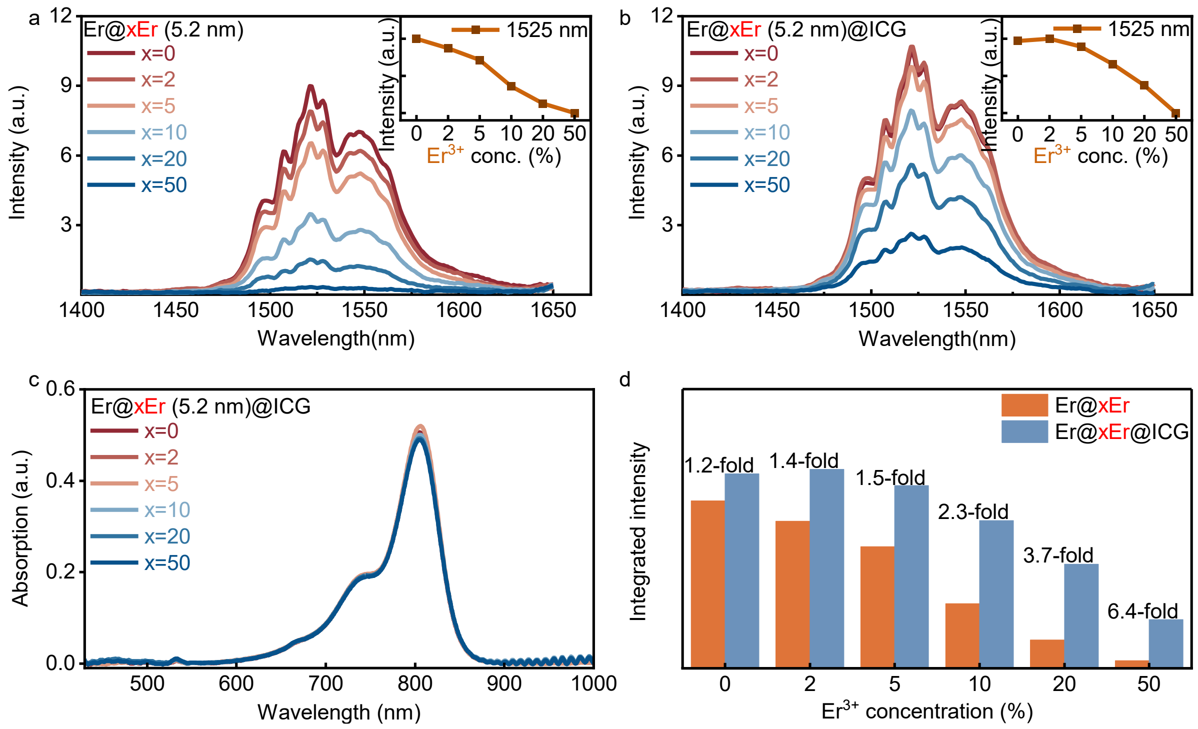

Figure S11. (a, b) DSL spectra of unsensitized and ICG-sensitized Er@xEr-5.2 nm (x = 0, 2, 5, 10, 20, 50%) under 808 nm excitation. (c) Absorption spectra of Er@xEr-5.2 nm@ICG with varying Er^{3+} doping concentrations (x = 0, 2, 5, 10, 20, 50%). According to the spectra, the dye-to-nanoparticle ratio is approximately 37. (d) Relevant quantitative statistics of emission intensities at 1525 nm from (a) and (b).

Figure S12. DSL spectra (with and without ICG) for Er@Y and Er@2Er nanoparticles with different shell thicknesses.

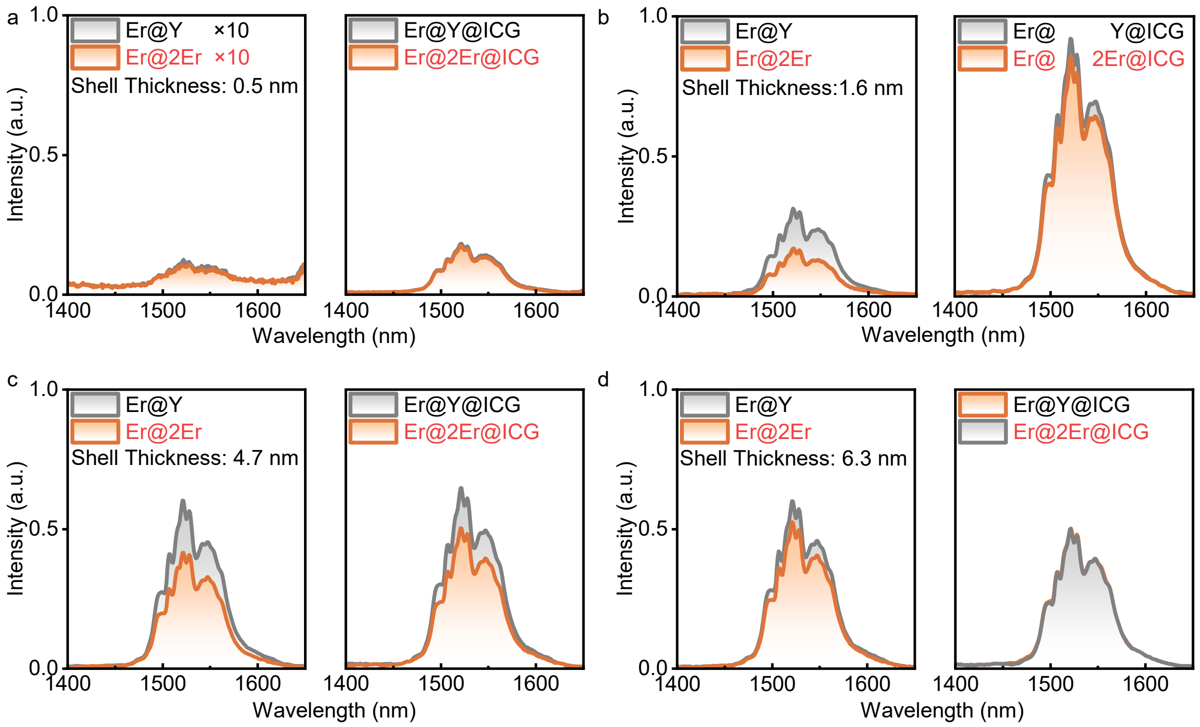

Figure S12. DSL spectra (with and without ICG) for Er@Y and Er@2Er nanoparticles with shell thicknesses of (a) 0.5 nm, (b) 1.6 nm, (c) 4.7 nm, and (d) 6.3 nm, respectively.

Figure S13. Emission spectra of unsensitized and ICG-sensitized Er@xYb-1.8 nm (x = 0, 20, 50, 75, 100%) under 980 nm excitation.

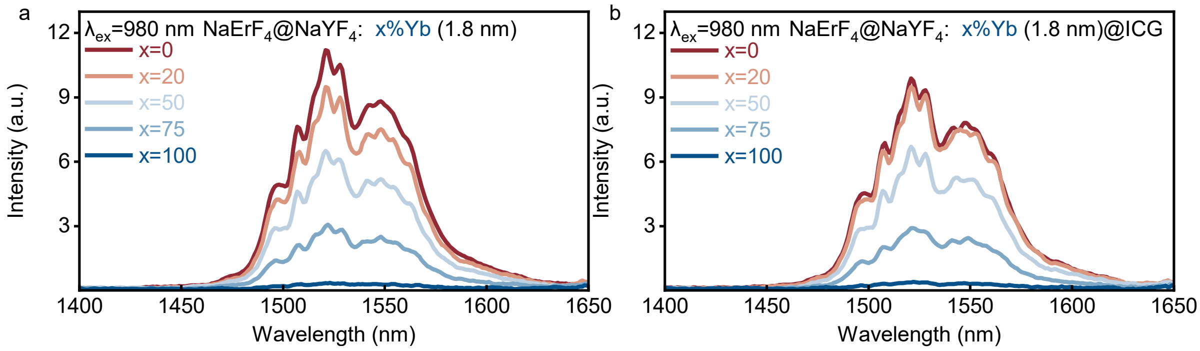

Figure S13. (a, b) DSL spectra of unsensitized and ICG-sensitized Er@xYb-1.8 nm (x = 0, 20, 50, 75, 100%) under 980 nm excitation.

Figure S14. Theoretical Model for Downshifting Luminescence and Energy Transfer From Yb^{3+} to Er^{3+} ($ET_{Yb \to Er}$).

The rate equation analysis provides a clear physical interpretation to identify further the dependence between downshifting luminescence and ET from Yb^{3+} to Er^{3+}. Generally, luminescence mechanisms in lanthanide systems with multiple electronic excited states include several processes, including ground state absorption (GSA), $ET_{Er \to Yb}$, $ET_{Yb \to Er}$, luminescence, and non-radiation relaxation (multiphonon relaxation and cross-relaxation). According to the spectra analyzed above and energy level diagrams of Er^{3+}, possible downshifting mechanisms are schematically illustrated in Figure S14, and we assume the possible model:

1) The ground-state population E_0 density is considered constant under low-excitation conditions.

2) The E_3, E_4 and E_5 excited states of Er^{3+} are pumped from GSA of $T_0 \rightarrow T_3$, $T_1 \rightarrow T_4$ and $T_2 \rightarrow T_5$ transition, respectively. Furthermore, their population is determined by the absorption ($\boldsymbol{\sigma}$) and the excitation power density ($\boldsymbol{\rho}$).

3) Under 808 nm excitation, the energy transfer from Er^{3+} to Yb^{3+} as $ET_{Er \to Yb}$, and the ET from Yb^{3+} to Er^{3+} are characterized by $\boldsymbol{\omega}$ and $\boldsymbol{\omega}_B$, respectively.

4) The excited states i of Er^{3+} have lifetimes τ_i and decay with rate constants $\alpha_i + \beta_i = \tau_i$, α_i, β_i represent radiative and non-radiative transition rates, respectively.

5) The excited state j of Yb^{3+} decays with rate constants $\gamma_j + \xi_j = \tau_j$, γ_j, ξ_j represent radiative and non-radiative transition rates, respectively.

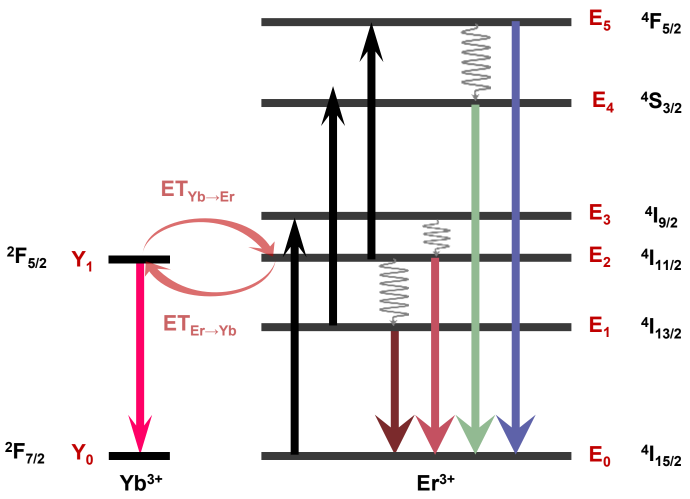

Figure S14. Simplified energy level structure showing the six-level system of Er@50Yb.

In the steady state achievable under continuous-wave (cw) excitation, all time derivatives are zero. Taking into account the eight-level system schematically depicted in Figure S14, the rate equations that include all the energy transfer processes described above are given by

$$\frac{dY_1}{dt} = \omega E_2 - (\gamma_1 + \delta_1 + \omega_B)Y_1 = 0 \qquad S_1$$

$$\frac{dE_1}{dt} = \beta_2 E_2 - (\alpha_1 + \beta_1 + \sigma\rho)E_1 = 0 \qquad S_2$$

$$\frac{dE_2}{dt} = \omega_B Y_1 + \beta_3 E_3 - (\alpha_2 + \beta_2 + \omega + \sigma\rho)E_2 = 0 \qquad S_3$$

$$\frac{dE_3}{dt} = \sigma\rho E_0 - (\alpha_3 + \beta_3)E_3 = 0 \qquad S_4$$

$$\frac{dE_4}{dt} = \sigma\rho E_1 + \beta_5 E_5 - (\alpha_4 + \beta_4)E_4 = 0 \qquad S_5$$

$$\frac{dE_5}{dt} = \sigma\rho E_2 - (\alpha_5 + \beta_5)E_5 = 0 \qquad S_6$$

Thus, the emission intensity $\boldsymbol{I}$ of $^4I_{13/2}$ level is given by

$$I = \alpha_1 E_1 = \frac{\alpha_1 \beta_2 \beta_3 \sigma \rho}{(\alpha_3+\beta_3)(\alpha_1+\beta_1+\sigma\rho)} \times \frac{1}{\frac{\omega(\gamma_1+\delta_1)}{\gamma_1+\delta_1+\omega_B}+\alpha_2+\beta_2+\sigma\rho} E_1 \qquad S_7$$

According to S_7, the enhancement part of the population of $^4I_{13/2}$ level with Yb^{3+} doping is determined by the $ET_{Er\to Yb}$ rate ($\boldsymbol{\omega}$) and $ET_{Yb\to Er}$ rate ($\boldsymbol{\omega}_B$) between Er^{3+} and Yb^{3+}.

Figure S15. Investigation of the Energy Transfer Mechanism via Triplet State Quenching.

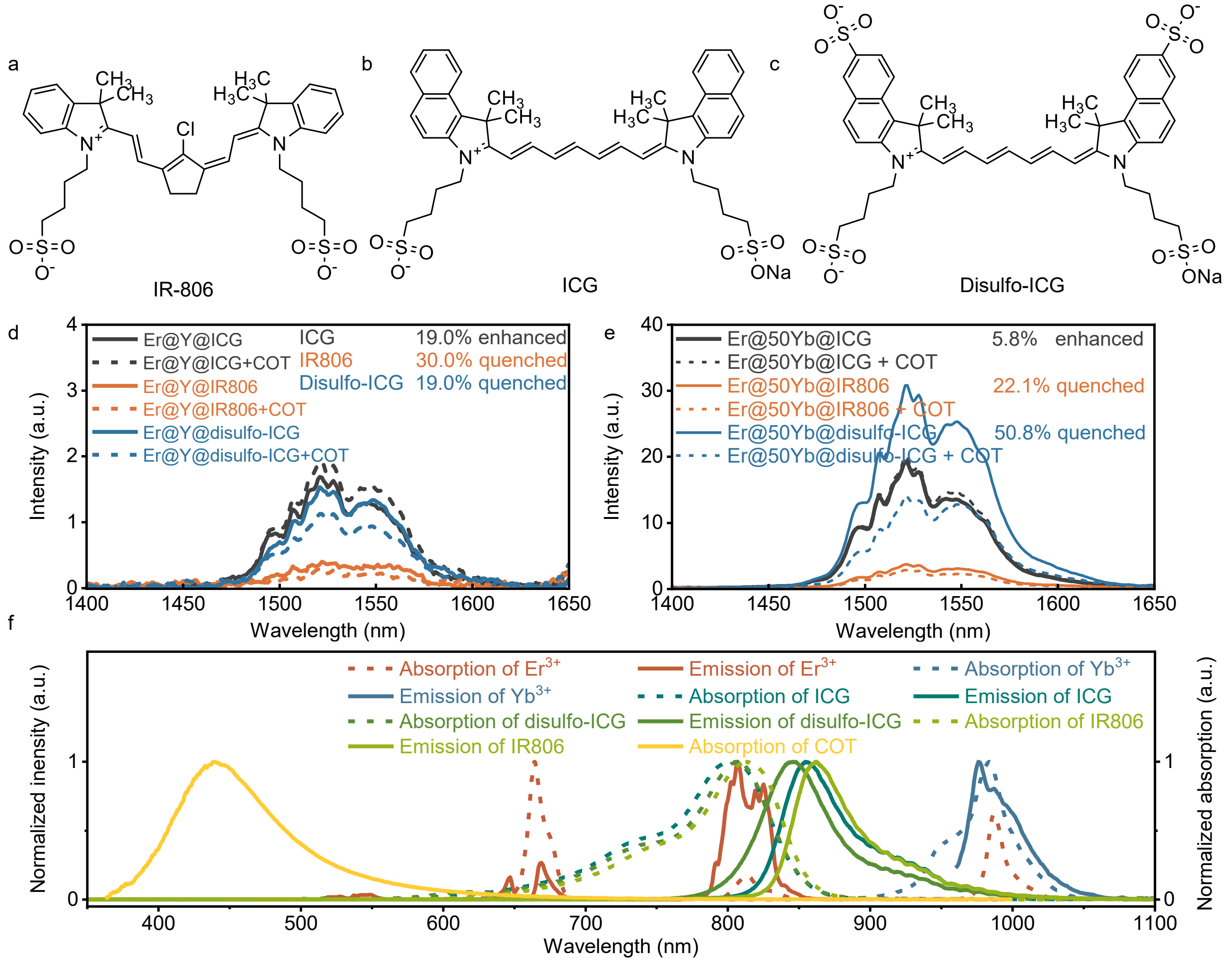

Figure S15. Chemical structures of (a) IR806, (b) ICG, and (c) disulfo-ICG. (d) DSL spectra of Er@Y@IR806, Er@Y@ICG and Er@Y@disulfo-ICG with and without the addition of triplet quencher COT under 808 nm excitation. (e) DSL spectra of Er@50Yb@IR806, Er@50Yb@ICG and Er@50Yb@disulfo-ICG with and without the addition of triplet quencher COT under 808 nm excitation. (f) Absorption spectra and emission spectra of Er^{3+}, Yb^{3+}, ICG, disulfo-ICG, and IR806, along with absorption spectra of COT.

Figure S16. Repeatability testing.

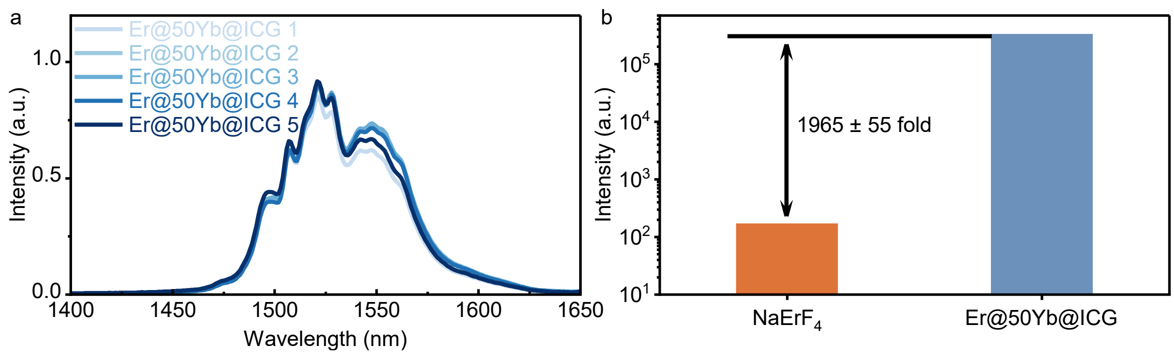

Figure S16. (a) The luminescence intensity of the Er@50Yb@ICG was measured five times. (b) the enhancement factor of Er@50Yb@ICG relative to $NaErF_4$ (mean ± s.d., n = 5).

Figure S17. Stability of Er@50Yb@ICG@DSPE-PEG_{2000} monitored by DLS.

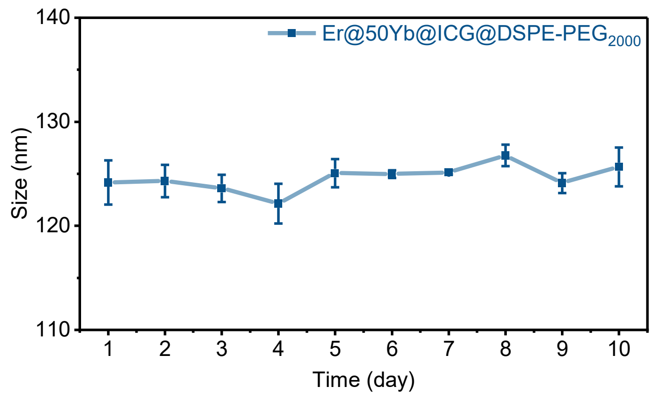

Figure S17. Hydrodynamic diameter of Er@50Yb@ICG@DSPE-PEG_{2000} during 10 days. The hydrodynamic diameter remained stable at 124.6 ± 1.7 nm throughout. The data represented mean ± SD, n = 3.

Figure S18. The photostability of Er@50Yb@ICG@DSPE-PEG_{2000}.

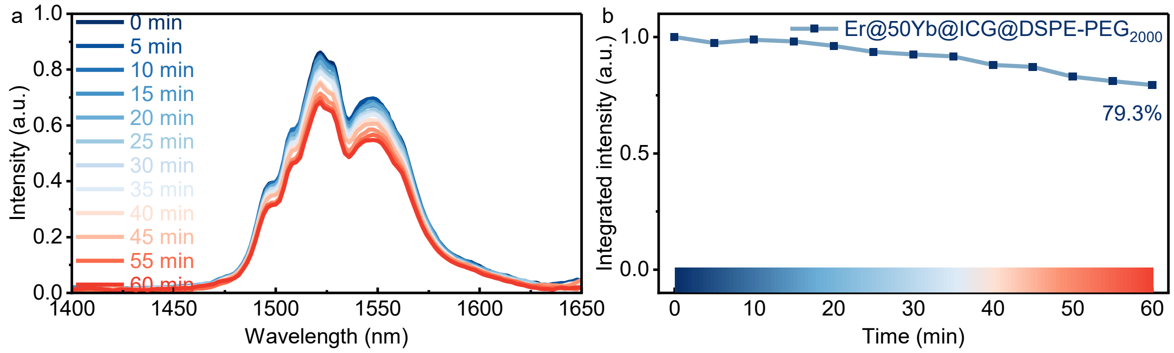

Figure S18. (a) Photostability of Er@50Yb@ICG@DSPE-PEG_{2000} under 808 nm laser. (a) Evolution of UCL

spectra over 60 min (330 mW cm^{-2}). (b) Corresponding decay of the integrated UCL intensity.

Figure S19. CCK8 Assay.

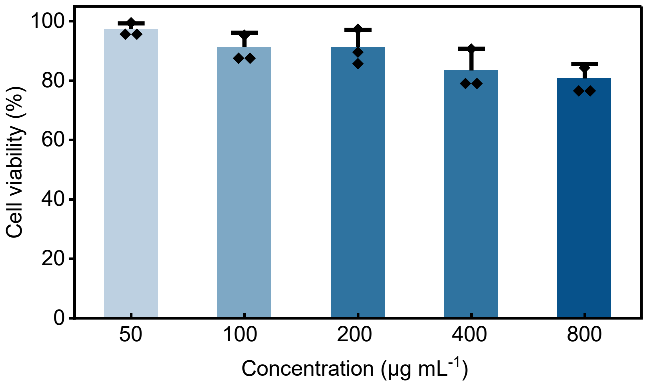

Figure S19. Cell viabilities of L929 cells treated with different concentrations of Er@50Yb@ICG@DSPE-PEG$_{2000}$ probe are presented as the mean ± standard deviation (n = 3).

Figure S20. Experiment on the Penetration Depth of NIR Lights.

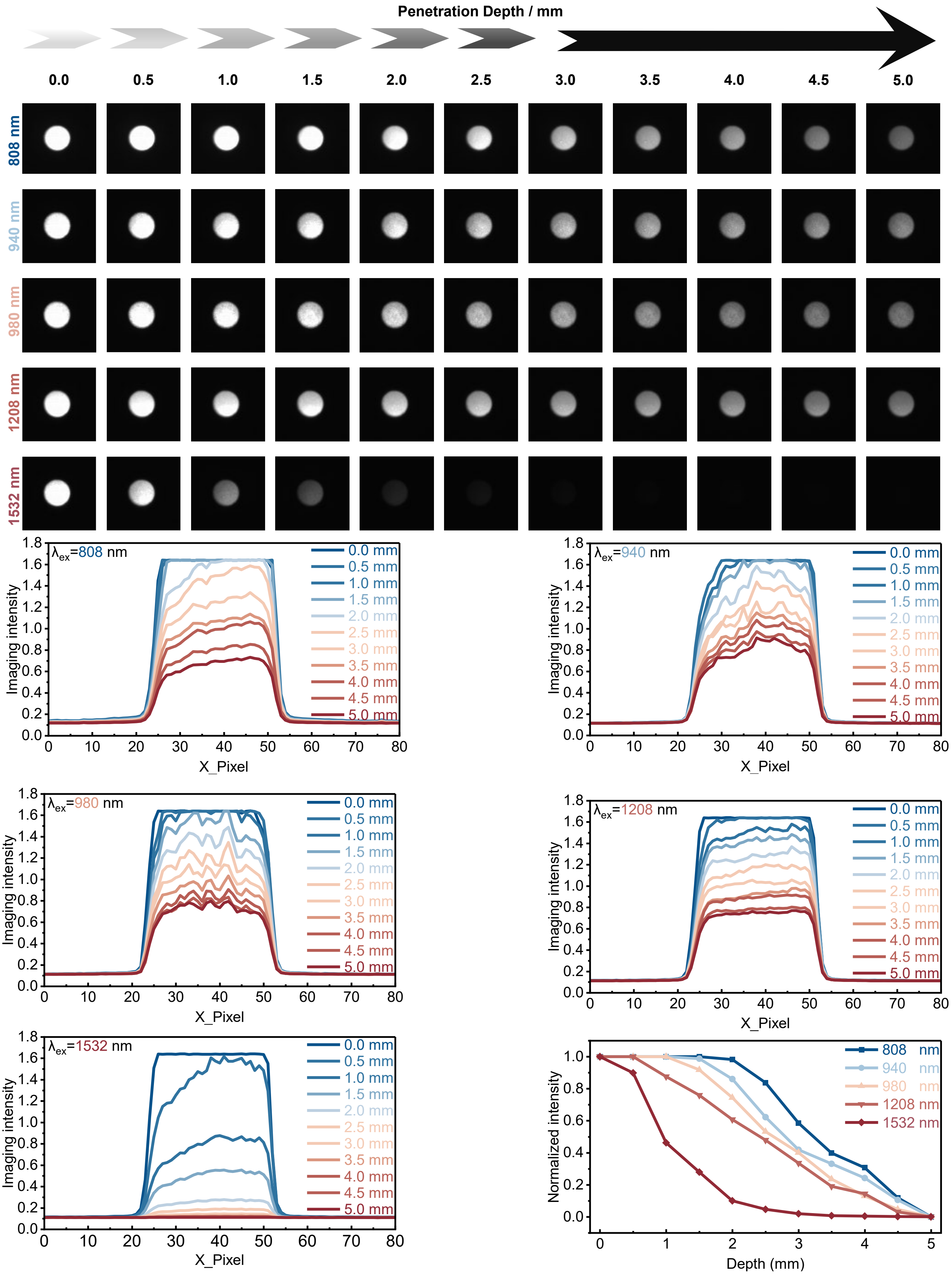

Figure S20. Comparison among various NIR lasers covered with 0-5 mm 1% intralipid solutions (808, 940,

980, 1208 and 1532 nm).

Figure S21. In vivo NIR-IIb imaging.

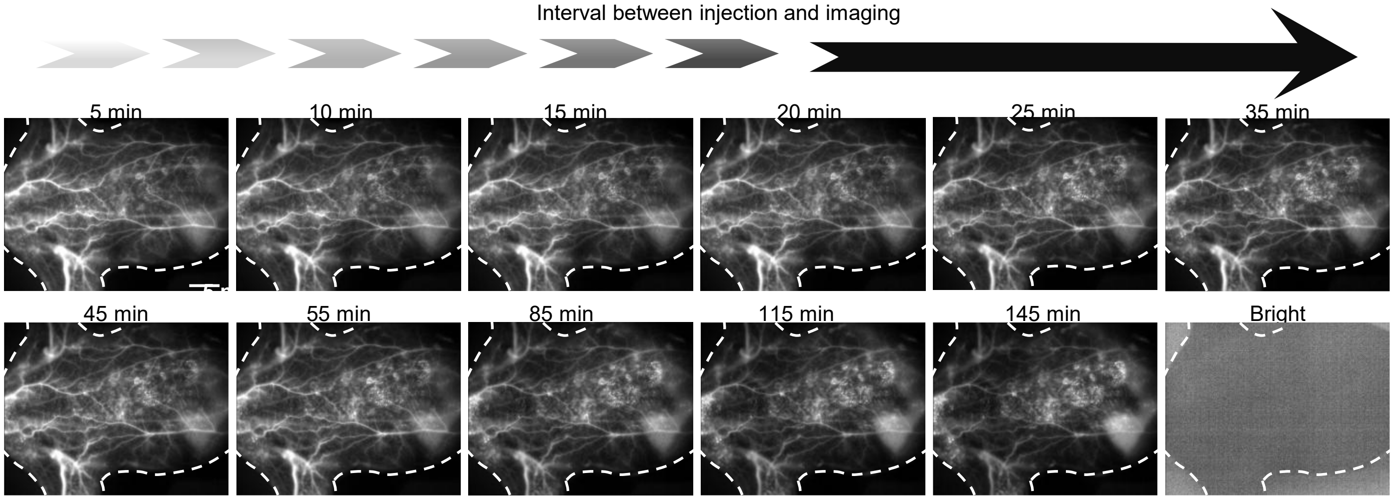

Figure S21. Non-invasive visualization of dynamic blood circulation in the NIR-IIb window. Sequential whole-body vascular NIR-IIb imaging under 808 nm excitation.

After intravenously injecting Er@50%Yb@ICG@DSPE-PEG$_{2000}$, imaging was performed upon 808 nm excitation (~113 mW cm^{-2}). The mice were anaesthetized and placed on the imaging stage. In vivo NIR-II imaging was carried out by using the InGaAs (SD 640, Tekwin, 50 ms high-gain model) with various filters (long-pass filters with a cut-on wavelength of 850 nm, 1000 nm, and 1100 nm were used, respectively).

Section 4. References

1. Chang, Y. L. et al. Bright Tm^{3+}-based downshifting luminescence nanoprobe operating around 1800 nm for NIR-IIb and c bioimaging. *Nature Communications* **14**, 1079 (2023).
2. Chen, Z. H. et al. An extended NIR-II superior imaging window from 1500 to 1900 nm for high-resolution *in vivo* multiplexed imaging based on lanthanide nanocrystals. *Angewandte Chemie International Edition* **62**, e202311883 (2023).
3. Guo, Y. W. et al. In vivo NIR-II fluorescence lifetime imaging of whole-body vascular using high quantum yield lanthanide-doped nanoparticles. *Small* **19**, 2300392 (2023).
4. Xu, R. et al. In vivo high-contrast biomedical imaging in the second near-infrared window using ultrabright rare-earth nanoparticles. *Nano Letters* **23**, 11203-11210 (2023).
5. Bao, H. Q. et al. Interfacial stress-modulated mechanosensitive upconversion luminescence of $NaErF_4$ based heteroepitaxial core–shell nanoparticles. *Advanced Optical Materials* **10**, 2101702 (2022).
6. Ashoka, A. H. et al. Brightness of fluorescent organic nanomaterials. *Chemical Society Reviews* **52**, 4525-4548 (2023).
